# Aurora: a fluorescent deoxyribozyme for high-throughput screening

**DOI:** 10.1093/nar/gkae467

**Published:** 2024-06-11

**Authors:** Martin Volek, Jaroslav Kurfürst, Matúš Drexler, Michal Svoboda, Pavel Srb, Václav Veverka, Edward A Curtis

**Affiliations:** Institute of Organic Chemistry and Biochemistry of the Czech Academy of Sciences, Prague 166 10, Czech Republic; Department of Genetics and Microbiology, Faculty of Science, Charles University in Prague, Prague 128 44, Czech Republic; Institute of Organic Chemistry and Biochemistry of the Czech Academy of Sciences, Prague 166 10, Czech Republic; Department of Informatics and Chemistry, University of Chemistry and Technology, Prague 166 28, Czech Republic; Institute of Organic Chemistry and Biochemistry of the Czech Academy of Sciences, Prague 166 10, Czech Republic; Institute of Organic Chemistry and Biochemistry of the Czech Academy of Sciences, Prague 166 10, Czech Republic; Institute of Organic Chemistry and Biochemistry of the Czech Academy of Sciences, Prague 166 10, Czech Republic; Institute of Organic Chemistry and Biochemistry of the Czech Academy of Sciences, Prague 166 10, Czech Republic; Department of Cell Biology, Faculty of Science, Charles University in Prague, Prague 128 44, Czech Republic; Institute of Organic Chemistry and Biochemistry of the Czech Academy of Sciences, Prague 166 10, Czech Republic

## Abstract

Fluorescence facilitates the detection, visualization, and tracking of molecules with high sensitivity and specificity. A functional DNA molecule that generates a robust fluorescent signal would offer significant advantages for many applications compared to intrinsically fluorescent proteins, which are expensive and labor intensive to synthesize, and fluorescent RNA aptamers, which are unstable under most conditions. Here, we describe a novel deoxyriboyzme that rapidly and efficiently generates a stable fluorescent product using a readily available coumarin substrate. An engineered version can detect picomolar concentrations of ribonucleases in a simple homogenous assay, and was used to rapidly identify novel inhibitors of the SARS-CoV-2 ribonuclease Nsp15 in a high-throughput screen. Our work adds an important new component to the toolkit of functional DNA parts, and also demonstrates how catalytic DNA motifs can be used to solve real-world problems.

## Graphical Abstract

**Graphical Abstract:**
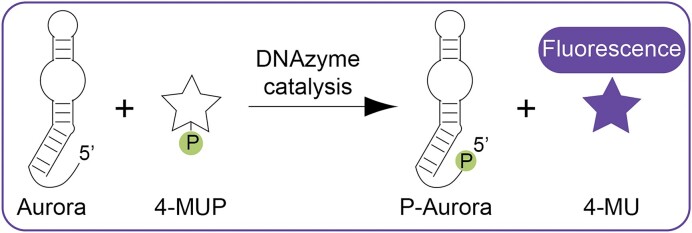

## Introduction

Fluorescence makes it possible to detect, visualize, and track molecules with high sensitivity and specificity. It also facilitates analysis of dynamic interactions important for molecular function. Fluorescence-based techniques are widely used in microscopy, immunology, cell sorting, DNA sequencing, diagnostics, and microarrays, and new applications continue to be developed. Such techniques offer a number of advantages relative to those with other types of readouts: they are typically more sensitive than colorimetric assays, offer greater flexibility and control over experimental readouts that chemiluminescent ones, and are safer than radioactive assays. One of the most powerful fluorescent tools is the fluorescent protein GFP (1,2). Originally identified in the jellyfish *Aequorea victoria*, fluorescent proteins have now been discovered in a wide range of organisms. The properties of these proteins have been enhanced by engineering, and variants have been developed that fold more efficiently, function over a wide range of conditions, and generate fluorescent signals with different colors. Such proteins have greatly facilitated studies of protein expression and localization. More recently, SELEX has been used to identify fluorescent RNA aptamers with a wide range of functional properties (3,4). These aptamers provide a way to investigate the functions of cellular RNA molecules, and engineered variants can also be used to monitor metabolite concentrations in real time. Although extremely useful for studies of biological systems, such motifs are less suitable for *in vitro* applications such as high-throughput screening. In the case of proteins this is related to both time and money: proteins are generally expensive and time-consuming to produce, and more difficult to evolve than nucleic acids. On the other hand, a significant limitation of fluorescent RNA aptamers is that they are unstable under many conditions due to the ubiquitous presence of ribonucleases (5).

DNA is another type of polymer capable of sophisticated functions, and functional DNA molecules such as aptamers and deoxyribozymes can be useful alternatives to functional protein and RNA motifs (5–8). DNA can be chemically synthesized at low cost, is stable over a wide range of conditions (including in the presence of ribonucleases, which are often present in samples), can typically be denatured and refolded without losing activity, and can be readily engineered using artificial evolution. Motifs with new functions (such as deoxyribozymes allosterically regulated by ligands) can be constructed by rational design or selection (9). And powerful enzymatic methods such as the polymerase chain reaction (PCR) (10) and rolling circle amplification (RCA) (11) can be used to copy and therefore amplify the signals generated by functional DNA. Despite these advantages, few methods to generate fluorescent signals using functional DNA motifs have been developed (12–17). One approach uses DNA aptamers that bind and enhance the fluorescence of ligands (12–14). The signal to noise ratios of these aptamers rarely exceed 100-fold, and tend to be significantly lower than those of their RNA counterparts (14). In addition, because the fluorophore must remain associated with the aptamer to generate a signal, this approach provides a less permanent and robust readout than a signal generated by a catalyst or enzyme. Another method utilizes a nonspecific peroxidase reaction catalyzed by DNA G-quadruplexes in the presence of hydrogen peroxide and a hemin cofactor (15,16). Although typically used to generate a colorimetric product, a fluorogenic signal can also be generated when this reaction is performed using phenolic substrates such as tyramine (17). Because the peroxidase reaction is also promoted by hemin itself, this method suffers from high background. Hydrogen peroxide is also incompatible with some types of assays, and high concentrations can inactivate the hemin cofactor. These limitations highlight the need for new and complementary methods to generate fluorescent signals using DNA.

In this study we used *in vitro* selection to identify a deoxyribozyme that generates a fluorescent signal by converting the coumarin substrate 4-MUP into the fluorescent product 4-MU (18). In a complementary study, a similar approach was employed to identify a deoxyribozyme that generates a colorimetric signal by converting the colorless substrate pNPP into the yellow product pNP (19). Our deoxyribozyme, which we named Aurora, offers a number of advantages relative to existing methods. Aurora works under mild conditions and uses an inexpensive and commercially available substrate. It is small, label free, and can be rapidly synthesized at low cost. Aurora is a potent enhancer of fluorescence, and generates a signal in minutes with a signal to noise ratio of >700. It is highly specific for its substrate and orthogonal to a chemiluminescent deoxyribozyme previously discovered in our group (20). This means that it could potentially be useful for multiplex applications (e.g. by first analyzing light production in the absence of excitation and then, after the signal has decayed, exciting the sample and analyzing fluorescence). Aurora can be modified to only generate fluorescence in the presence of an input of interest (such as a target molecule in a sample). It is also useful for real-world applications: an engineered variant can detect ribonuclease activity with a limit of detection of ∼100 pM, and was used to identify small molecule inhibitors of the SARS-CoV-2 ribonuclease Nsp15 in a high-throughput screen. Our results provide a new and improved way to construct fluorescent sensors using DNA. They also show how such sensors can be used to solve real-world problems.

## Materials and methods

### Oligonucleotides

Oligonucleotides were chemically synthesized by GENERI BIOTECH s.r.o., Sigma-Aldrich or IDT and purified by PAGE or HPLC. See Supplementary Table S1 for the sequences of all oligonucleotides used in this study.

### Pool design

The library used in our initial selection (Pool 1 in Supplementary Table S1) was generated by randomly mutagenizing the H1 variant of Supernova (a chemiluminescent deoxyribozyme recently discovered in our group (20)) at a rate of 21% per position. A 20-nucleotide long primer-binding site was also added to the 3′ end. The library used for the reselection (Pool 2 in Supplementary Table S1) was based on the sequence of Hit10. This generated fluorescence with the highest signal to noise ratio of any of the deoxyribozymes we tested from the initial selection. The 85 positions in Hit10 were randomly mutagenized at a rate of 21% per position and a new 20-nucleotide long primer-binding site was added to the 3′ end.

### Initial selection

The single-stranded DNA pool (Pool1) and blocking oligonucleotide (REV1) were mixed in Milli-Q water. After heating at 65°C for 2 min and cooling at room temperature for 5 min, 5× selection buffer and then the disodium salt of the 4-methylumbelliferyl phosphate substrate (4-MUP) were added. Final concentrations were 1 μM Pool1, 1.5 μM REV1, 1× selection buffer (200 mM KCl, 1 mM ZnCl_2_, 1 μM Ce(NO_3_)_4_, 0.1 μM PbCl_2_, 50 mM HEPES pH 7.4) and 1 mM 4-MUP. After incubating for 2.4 h, DNA was concentrated by ethanol precipitation. A short oligonucleotide (FWD1) was then ligated to library members containing a 5′ phosphate. To increase the efficiency of the ligation, the reaction was performed in the presence of a splint oligo (Splint1) which was complementary to both FWD1 and the 5′ end of Pool1. The ligation reaction was incubated for 5 min at 37°C. Final concentrations were 2.5 μM Pool1, 2.5 μM FWD1, 2.5 μM Splint1, 1 × T4 DNA ligase buffer and 0.5 Weiss units of T4 DNA ligase per 1.0 μg of Pool1. DNA molecules were then separated by 6% urea–PAGE and DNA molecules that co-migrated with a 125-nucleotide marker were cut from the gel, eluted and ethanol precipitated. They were then amplified by PCR using Q5 HotStart DNA Polymerase and the FWD1r and REV1p primers. Final concentrations were 500 × diluted Pool1, 0.5 μM FWD1r, 0.5 μM REV1p, 1 × Q5 reaction buffer, 1 × Q5 high GC enhancer, 0.2 mM dNTPs and 0.02 U of Q5 HotStart DNA polymerase per 1 μl of the PCR reaction mixture. Double-stranded PCR products were isolated using a Macherey-Nagel PCR Clean-up kit. The reverse primer REV1p contained a 5′ phosphate, and the strand synthesized using this primer (which was complementary to Pool1) was digested using λ-exonuclease. Final concentrations were 5 μg of the double-stranded PCR product, 1 × Lambda Exonuclease reaction buffer and 1 μl (5 U) of Lambda Exonuclease in a volume of 50 μl. The Lambda Exonuclease mixture was incubated at 37°C for 1 h. The resulting single-stranded DNA molecules (of length 125 nucleotides) were purified using a Macherey-Nagel PCR Clean-up kit. The FWD1r primer used in the PCR contained a single RNA linkage at its 3′ end. This made it possible to regenerate the 5′ end of the Pool1 by base hydrolysis. To do this, DNA was heated at 65°C for 2 min, cooled at room temperature for 5 min, and mixed with 10 × hydrolysis buffer (1 × hydrolysis buffer: 20 mM Trizma base, 400 mM KOH, 4 mM EDTA). The RNA linkage was then base hydrolyzed at 90°C for 10 min. The resulting 105 nucleotide long DNA molecules (corresponding to single-stranded Pool 1 molecules with a 5′ hydroxyl group) were then isolated by 6% urea–PAGE and ethanol precipitation. After the fifth round of selection the library was amplified by PCR, purified using a Macherey-Nagel PCR Clean-up kit, and sequenced by Eurofins Genomics using an amplicon paired-end sequencing run.

### Reselection

Reselection conditions were the same as those used in the initial selection except for the following differences. First, Pool2 was used instead of Pool1. Second, the library was incubated with 4-MUP for 14.4 min rather than 2.4 h. Third, a new blocking oligonucleotide and reverse primer (REV2/REV2p) was used (Supplementary Table S1). This library was sequenced after the sixth round by Eurofins Genomics using an amplicon paired-end sequencing run.

### Analysis of fluorescence production

Oligonucleotides corresponding to individual sequences from evolved libraries were ordered from GENERI BIOTECH s.r.o. Fluorescence production was measured as follows: oligonucleotides were re-suspended in Milli-Q water, heated at 65°C for 2 min, and cooled at room temperature for 5 min. After adding 5 × selection buffer or 5 × Aurora buffer, samples were transferred to a white half-area 96-well plate (Corning). 4-MUP was then added. In continuous assays, fluorescence was measured for 4 h using a Tecan Spark plate reader (Tecan Group). In discontinuous assays, after incubating for a specific time, samples were quenched with 20 μl of 1 M KOH and fluorescence was measured using a plate reader. In a typical experiment final concentrations were 15 μM of the tested oligonucleotide and either 1 × selection buffer (200 mM KCl, 1 mM ZnCl_2_, 1 μM Ce(NO_3_)_4_, 0.1 μM PbCl_2_, 50 mM HEPES pH 7.4) or 1 × Aurora buffer (200 mM KCl, 1 mM ZnCl_2_, 50 mM HEPES pH 7.4, 5% (v/v) DMSO) and 30 μM 4-MUP. Fluorescence was measured in a white half-area 96-well plate (Corning) using a Tecan Spark plate reader with the following settings: excitation 358 (±5) nm, emission 455 (±5) nm, 97 nm wavelength gap, optimal gain, 30 flashes, Z position calculated from one well in the plate.

### Analysis of phosphorylation

Oligonucleotides corresponding to individual sequences from evolved libraries were ordered from GENERI BIOTECH s.r.o., purified by 6% urea–PAGE or HPLC, and resuspended in Milli-Q water. Self-phosphorylation reactions were performed by first heating deoxyribozymes at 65°C for 2 min and cooling at room temperature for 5 min. After mixing with 5 × selection buffer or 5 × Aurora buffer, the 4-MUP substrate was added. Final concentrations in a typical reaction were 1 μM deoxyribozyme, 1 × selection buffer (200 mM KCl, 1 mM ZnCl_2_, 1 μM Ce(NO_3_)_4_, 0.1 μM PbCl_2_, 50 mM HEPES pH 7.4) or 1 × Aurora buffer (200 mM KCl, 1 mM ZnCl_2_, 50 mM HEPES pH 7.4, 5% (v/v) DMSO), and 1 mM 4-MUP unless stated otherwise. Reactions were incubated for specific times at room temperature and stopped by the addition of EDTA to a final concentration of 25 mM. Reactions were then concentrated by ethanol precipitation, and reacted deoxyribozymes (now containing a 5′ phosphate) were ligated to a short oligonucleotide as described in the section ‘Initial Selection.’ Reacted and unreacted molecules were separated by 6% urea–PAGE. DNA was visualized by staining with GelRed using the protocol recommended by the manufacturer. Gels were scanned using a Typhoon laser scanner and the percentage of reacted and unreacted molecules was quantified using ImageQuant TL software.

### Calculation of signal to noise ratios

Signal to noise ratios were defined as the fluorescence of a sample in the presence of deoxyribozyme divided by the fluorescence of the sample in the absence of the deoxyribozyme. The background signal was defined as the fluorescence of 1 × Aurora buffer (200 mM KCl, 50 mM HEPES, pH 7.4, 1 mM ZnCl_2_ and 5% (v/v) DMSO) and was subtracted before calculating signal to noise ratios.

### Optimization of reaction conditions

To maximize fluorescence we searched for optimal reaction conditions. The optimal DNA, 4-MUP, KCl, ZnCl_2_ and HEPES concentrations were determined by titration experiments. We also tested the effects of different monovalent and divalent metal ions, an organic solvent (DMSO), and a molecular crowding agent (PEG 200) on activity. Titration experiments to determine the optimal pH during and after the reaction were also performed. Aurora 2 (Supplementary Table S1) was used for these experiments if not stated otherwise. Activity was measured by analysis of fluorescence production (using a plate reader assay) and self-phosphorylation (using a ligation assay).

### Kinetic measurements and analysis

Kinetic measurements were performed using a ligation assay. Deoxyribozyme (either Aurora 1 or Aurora 2; Supplementary Table S1) was mixed with Milli-Q water, heated at 65°C for 2 min, and cooled at room temperature for 5 min. 5 × Aurora buffer and 4-MUP were then added. Final concentrations were 1 μM deoxyribozyme, 1 × Aurora buffer (200 mM KCl, 1 mM ZnCl_2_, 50 mM HEPES pH 7.4, 5% (v/v) DMSO) and 1 μM to 300 μM 4-MUP. Reactions were incubated for specific times at room temperature and stopped by the addition of EDTA to a final concentration of 25 mM. Reactions were stopped at time points that corresponded to the linear phase of the reaction. After ethanol precipitation, reacted deoxyribozyme (containing a 5′ phosphate) was ligated to a short oligonucleotide as described in the section ‘Initial Selection’. Reacted and unreacted molecules were separated by 6% urea–PAGE. DNA was visualized by staining with GelRed using the protocol recommended by the manufacturer and gels were scanned using a Typhoon laser scanner. The percentage of reacted and unreacted deoxyribozyme was quantified using ImageQuant TL software. *k*_cat_ and *K*_m_ values were obtained using Prism 9 software. Curves were fit using the equations: *V*_0_ = *V*_max_ × [S]/(*K*_m_ + [S]) to obtain *K*_m_ and *k*_cat_= *V*_max_/[E] to obtain *k*_cat_.

### Next generation sequencing and data analysis

All libraries were sequenced by Eurofins Genomics using amplicon paired-end sequencing runs. Raw reads were processed using a pipeline consisting of adaptor trimming (cutadapt v1.18), read merging (fastq-join v1.3.1), unifying of read orientation (fastx barcode splitter), primer clipping (cutadapt v1.18), length filtering (cutadapt v1.18) and counting of unique sequences (bash). All further analysis was performed using in house python scripts available at https://github.com/Jardic/aurora_selection_analysis.

### Oligonucleotide detection using an engineered version of Aurora

The oligonucleotide sensor was mixed with the target oligonucleotide in water, heated at 98°C for 2 min, and immediately cooled on ice for 5 min. 5 × Aurora buffer and DMSO were then added. Samples were transferred to a white half-area 96-well plate (Corning), 4-MUP was added, and the reaction mixture was incubated for 4 h at room temperature. Final concentrations were 5 μM of the oligonucleotide sensor, 10 μM of the target oligonucleotide, 1 × Aurora buffer (200 mM KCl, 50 mM HEPES pH 7.4, 1 mM ZnCl_2_ and 5% (v/v) DMSO) and 30 μM 4-MUP. After 4 h the reaction was stopped by adding 20 μl of 1 M KOH, and fluorescence was then measured using a Tecan Spark plate reader. Analysis of fluorescence production was performed as described below in ‘Calculation of signal to noise ratios’.

### RNase A sensor based on Aurora

The RNase A sensor was heated at 65°C for 2 min, and cooled at room temperature for 5 min. Then 5 × Aurora buffer and DMSO were added. Samples were transferred to a white half-area 96-well plate (Corning), and 4-MUP and either RNase A (Thermo Fisher Scientific) alone or RNase A and RiboLock (Thermo Fisher Scientific) were added. The reaction mixture was incubated for 4 h at room temperature. Final concentrations were 5 μM of the RNase A sensor, 500 nM RNase A or 500 nM RNase A plus 500 nM RiboLock, 1 × Aurora buffer (200 mM KCl, 50 mM HEPES pH 7.4, 1 mM ZnCl_2_ and 5% (v/v) DMSO) and 30 μM 4-MUP if not stated otherwise. After 4 h the reaction was stopped by adding 20 μl of 1 M KOH to the reaction mixture. Fluorescence was then measured using a Tecan Spark plate reader. Analysis of fluorescence production was performed as described below in the section ‘Calculation of signal to noise ratios’.

### Plasmid construction, expression, and purification of Nsp15 from SARS-CoV-2

Nsp15 cloning, expression and purification were performed as described in Kim *et al.* (21) with minor modifications. A synthetic DNA sequence encoding an *Escherichia coli* codon optimized version of Nsp15 was cloned into a pMCSG7 vector using Gibson assembly. Cloning was confirmed by Sanger sequencing. The final pSARS-CoV-2-Nsp15_6 × His vector encoded the full-length Nsp15 protein fused to an N-terminal hexahistidine tag via a TEV protease cleavage site. *E. coli* NiCo21(DE3) cells (New England Biolabs) were transformed with this plasmid. For large-scale expression and purification, a 3 l culture of LB medium was grown at 37°C in a LEX bioreactor (Epiphyte3) in the presence of 100 μg/ml ampicillin. Once the culture reached OD_600_ ∼ 1.0, flasks were moved to an 18°C bioreactor bath and supplemented with 0.1% glucose and 40 mM K_2_HPO_4_ (final concentration). Protein expression was induced by the addition of 0.2 mM IPTG for 16 h at 18°C. Bacterial cells were harvested by centrifugation at 7000g and cell pellets were resuspended in 40 ml lysis buffer (50 mM HEPES, 500 mM NaCl, 5% [v/v] glycerol, 20 mM imidazole, 10 mM β-mercaptoethanol, pH 8.0) per liter of culture and lysed using a CF1 high-pressure homogenizer. Cellular debris was removed by centrifugation at 25 000g for 40 min at 4°C. The supernatant was filtered through a 0.45 μm filter, mixed with 2 ml of Ni^2+^ Sepharose equilibrated with lysis buffer, and the suspension was added to a gravity-flow column. Unbound proteins were removed by washing with 40 ml of lysis buffer. Bound proteins were eluted with 10 ml of lysis buffer supplemented with 500 mM imidazole pH 8.0. A final purification was performed using a Superdex 200 column equilibrated in lysis buffer in which 10 mM β-mercaptoethanol was replaced by 1 mM TCEP. Fractions containing Nsp15 were collected. Lysis buffer was replaced with storage buffer (150 mM NaCl, 20 mM HEPES pH 7.5, 1 mM TCEP) via repeated concentration and dilution using a 30 kDa MWCO filter (Amicon-Millipore). The final protein sample was concentrated to 1 mg/ml, aliquoted, snap frozen in liquid nitrogen and stored at –80°C until further use.

### High-throughput screen for Nsp15 inhibitors using an Aurora Nsp15 sensor

Small molecules from a 1000-member fragment screen library (Maybridge) were transferred to the wells of 384-well plates using an Echo 550 liquid handler. Nsp15 protein in 1 × Nsp15 buffer (50 mM KCl, 20 mM HEPES pH 7.4, 5 mM MnCl_2_, 0.003% (v/v) Tween20) was then added using a CERTUS Flex liquid handler. After mixing, the Aurora Nsp15 sensor (in 1 × Nsp15 buffer) was added using a CERTUS Flex liquid handler. Reactions were mixed again. Final concentrations were 25 μM Aurora Nsp15 sensor, 400 nM Nsp15 protein, 1 × Nsp15 buffer (50 mM KCl, 20 mM HEPES pH 7.4 and 5 mM MnCl_2_), 0.003% (v/v) Tween20, and 200 μM small molecule from the fragment screen library in a volume of 20 μl. After incubating at room temperature for 1 h to allow Nsp15 to cleave and activate the Aurora Nsp15 sensor, 80 μl of 1 × Aurora reaction mixture (50 mM KCl, 20 mM HEPES pH 7.4, 1.25 mM ZnCl_2_, 6.25% (v/v) DMSO and 18.75 μM 4-MUP) was added using a CERTUS Flex liquid handler. The ZnCl_2_ in this buffer inhibited the Nsp15 protein while activating Aurora for catalysis. Final concentrations were 5 μM Aurora Nsp15 sensor, 80 nM Nsp15 protein, 40 μM small molecule from the fragment screen library, 1 × Aurora/Nsp15 buffer (50 mM KCl, 20 mM HEPES pH 7.4, 1 mM ZnCl_2_ 1 mM MnCl_2_), 0.0006% Tween20, 5% (v/v) DMSO, and 15 μM 4-MUP in a volume of 100 μl. The reaction mixture was incubated at room temperature for 4 h, and fluorescence was measured using a Tecan Spark plate reader. Fluorescence was measured in a black flat bottom 384-well plate (Corning). Analysis of fluorescence production was performed as described in the section ‘Calculation of signal to noise ratios’. A counter screen was also performed to confirm that the inhibitors identified in the initial screen inhibit the Nsp15 protein rather than Aurora. The counter screen was performed as described above, but Aurora 2 was used instead of the Aurora Nsp15 sensor.

### High-throughput screen for Nsp15 inhibitors using a FRET assay

Small molecules from a 1000-member fragment screen library (Maybridge) were transferred to the wells of 384-well plates using an Echo 550 liquid handler. Nsp15 protein in 1 × Nsp15 buffer (50 mM KCl, 20 mM HEPES pH 7.4, 5 mM MnCl_2_, 0.003% (v/v) Tween20) was then added using a CERTUS Flex liquid handler. After mixing, the FRET substrate (5′-FAM-AAArUAA-BHQ1-3′) in 1 × Nsp15 buffer was added using a CERTUS Flex liquid handler. Reactions were mixed again. Final concentrations were 25 μM FRET substrate, 400 nM Nsp15 protein, 1 × Nsp15 buffer (50 mM KCl, 20 mM HEPES pH 7.4 and 5 mM MnCl_2_), 0.003% (v/v) Tween20, and 200 μM small molecule from the fragment screen library in a volume of 20 μl. Fluorescence was measured every 20 min for 1 h in a black flat bottom 384-well plate (Corning) using a Tecan Spark plate reader with the following settings: excitation 485 (±5) nm, emission 527 (±5) nm, gain 134, 30 flashes, Z position calculated from the well.

### Analysis of data from high-throughput screens

Wells containing 1 mM ZnCl_2_ (which inhibited Nsp15 at this concentration) served as negative controls, and were used to determine background levels of fluorescence. The average value of this background was subtracted from the fluorescence values obtained from all other wells. Wells containing aliquots of DMSO alone rather than DMSO plus small molecule were used as positive controls. After subtraction of the background, the average value of these positive controls was defined as 100% Nsp15 activity. Activity of Nsp15 in the presence of small molecules from the fragment screen library was calculated relative to this positive control value. *Z-*factors (22) were calculated for each 384-well plate to determine the quality of the screen. *Z*-factors were calculated using the equation *Z*-factor = 1 – [3(*σ*_p_ + *σ*_n_)] / (*μ*_p_ - *μ*_n_) where *μ*_p_ is the mean fluorescence of the positive control, *μ*_n_ is the mean fluorescence of the negative control, *σ*_p_ is the standard deviation of the mean fluorescence of the positive control, and *σ*_n_ is the standard deviation of the mean fluorescence of the negative control.

### Calculation of IC50 values

IC50 values were measured for small molecules that strongly inhibited Nsp15 in high-throughput screens. Solutions containing different concentrations of these inhibitors were transferred to the wells of 384-well plates using an Echo 550 liquid handler. To obtain the same volume in each well, the drops containing small molecules were backfilled with DMSO to 200 nl. For each inhibitor characterized, IC50 values were measured using both the Aurora Nsp15 sensor and using the FRET assay. After determining the relative activity of Nsp15 at each concentration of inhibitor, IC50 values were calculated using Prism 9 software (GraphPad).

### NMR experiments

HPLC-purified DNA was purchased from GENERI BIOTECH s.r.o. DNA was re-suspended in Milli-Q water, heated at 65°C for 2 min, cooled at room temperature for 5 min, and 5 × Aurora buffer was then added. Concentrations at this point were 15 μM DNA, 200 mM KCl, 50 mM HEPES, pH 7.4 and 1 mM ZnCl_2_. Samples were concentrated using Ultra-Amicon Centrifugal Filter Units (cutoff 3 kDa) to 500 μM DNA and a 1.5 molar excess of 4-MUP, D_2_O and DSS were added. Final concentration were 500 μM DNA, 200 mM KCl, 50 mM HEPES, pH 7.4, 1 mM ZnCl_2_, 750 μM 4-MUP, 10% (v/v) D_2_O and a trace amount of DSS. NMR experiments were performed on a Bruker Avance III HD 850 MHz system equipped with an inverse triple resonance cryo-probe. Spectral analyses were performed using TOPSPIN (Bruker) software (23).

## Results and discussion

### Discovery of the fluorescent deoxyribozyme Aurora

**Figure 1.**
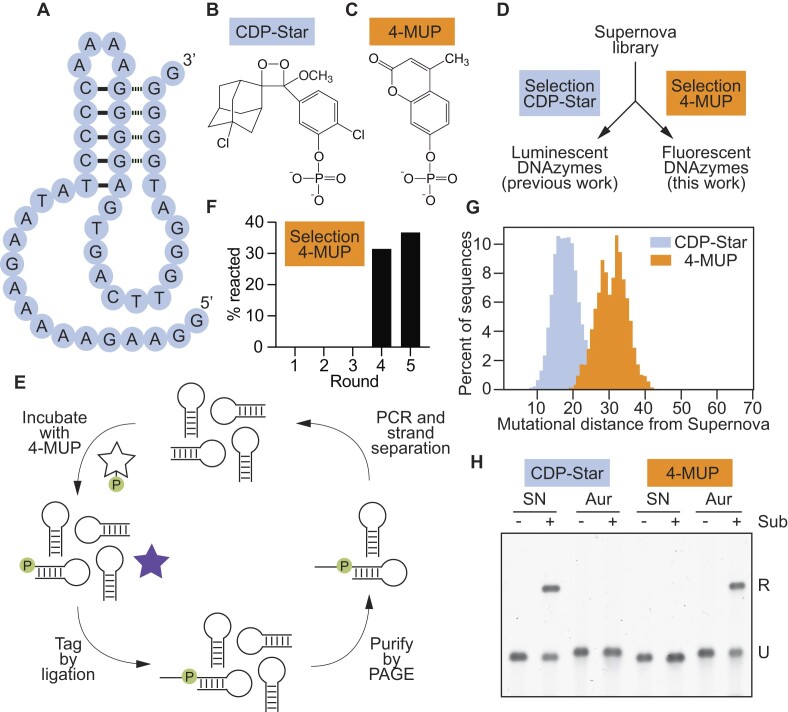
Identification of deoxyribozymes that generate fluorescence. (**A**) Secondary structure of Supernova, a chemiluminescent deoxyribozyme previously isolated in our group. (**B**) Chemical structure of CDP-Star, the substrate used by Supernova. (**C**) Chemical structure of 4-MUP, the substrate used in this study. (**D**) Workflow of a previous selection (in which deoxyribozymes that react with the original CDP-Star substrate were isolated from a library of variants of Supernova) and the selection performed here (in which deoxyribozymes that react with the substrate 4-MUP were isolated from the same library). (**E**) Artificial evolution protocol to identify deoxyribozymes that phosphorylate themselves in the presence of 4-MUP. (**F**) Progress of the selection for deoxyribozymes that can react with 4-MUP. (**G**) Distribution of mutational distances of sequences in a library of Supernova variants relative to Supernova itself after selection for the ability to react with CDP-Star (blue) or 4-MUP (orange). (**H**) The substrate specificities of Supernova and Aurora are orthogonal. Supernova (labeled ‘SN’) and Aurora 1 (labeled ‘Aur’) were each incubated separately with CDP-Star (left) or 4-MUP (right). Time points were analyzed using the ligation assay. See Supplementary Table S1 for the sequence of Aurora 1.

**Figure 2.**
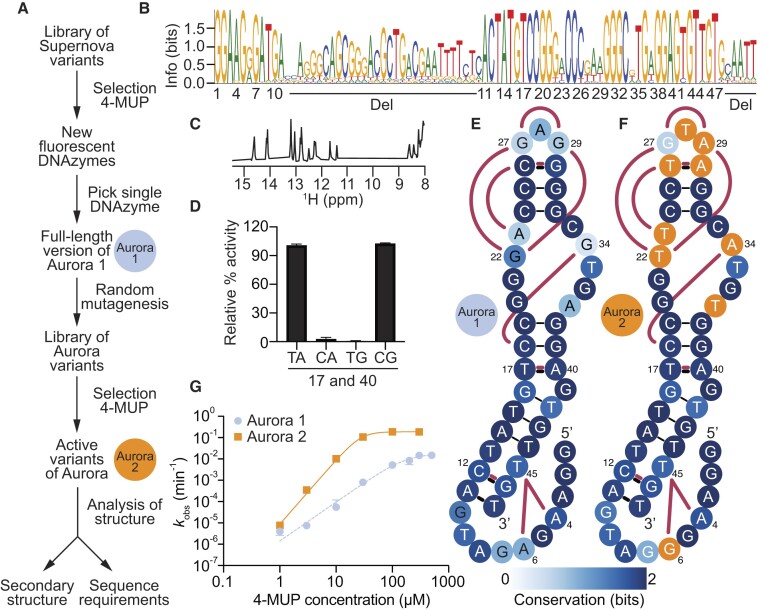
Sequence requirements and secondary structure of the fluorescent deoxyribozyme Aurora. (**A**) Evolutionary lineage of Aurora 1 (the minimized catalytic core of the initial isolate of Aurora) and Aurora 2 (an optimized variant isolated from a randomly mutagenized library based on Aurora 1 full-length). (**B**) Sequence logo generated from analysis of variants of Aurora using high-throughput sequencing. (**C**) Proton NMR spectrum of the 17C 40G variant of Aurora (Supplementary Table S1) showing chemical shifts consistent with base pairs. (**D**) Double-mutant cycle showing that positions 17 and 40 interact in a way that is consistent with base pairing. (**E**) Secondary structure model of Aurora 1. Base pairs are shown using solid black lines, interactions supported by mutual information analysis are shown in maroon, and the degree of conservation at each position is indicated by blue shading. (**F**) Secondary structure model of Aurora 2. Positions that differ from Aurora 1 are shown in orange. (**G**) Catalytic activity of Aurora 1 and 2 over a range of 4-MUP concentrations as measured using a ligation assay.

Supernova is a deoxyribozyme recently discovered in our laboratory (Figure 1A) (20). It transfers the phosphate group from the 1,2-dioxetane substrate CDP-Star (Figure 1B) to its own 5′ hydroxyl group, which triggers a chemically initiated electron exchange luminescence reaction and a flash of blue light (24–26). Deoxyribozymes that use substrates which generate orthogonal signals when they are dephosphorylated would bring new functionality to the toolkit of functional DNA parts. An example of such a substrate is the coumarin 4-MUP (Figure 1C) (18). Dephosphorylation of 4-MUP yields the fluorescent compound 4-MU (Table 1) (27,28), and a deoxyribozyme that promotes this reaction could in principle be used to generate a fluorescent signal. To search for such a deoxyribozyme, a library was generated by randomly mutagenizing the sequence of Supernova at a rate of 21% per position (Figure 1D). We used Supernova as the starting point for our library because this deoxyribozyme catalyzes a phosphoryl transfer reaction using a substrate with some similarities to 4-MUP (compare Figure 1B and C). After incubating with 4-MUP, library members containing a 5′ phosphate group were tagged by ligation, purified by PAGE, and amplified by PCR (Figure 1E). After four rounds of selection activity was detected (Figure 1F), and after one more round the library was characterized by high-throughput sequencing. Sequences from the evolved library with high read numbers could phosphorylate themselves in the presence of 4-MUP and also generate fluorescence (Supplementary Figure S1 and Supplementary Table S1). However, most appeared to be structurally and functionally distinct from Supernova (see also reference (29)). We initially appreciated this point by comparing the mutational distances of sequences from Supernova in a library separately challenged with two different substrates (Figure 1D). When a selection was previously performed to identify variants in this library that used CDP-Star (i.e. the original substrate) with improved efficiency (20), the average mutational distance of sequences in the evolved pool from Supernova was 18.42 (Figure 1G, blue peak). In contrast, the average mutational distance of variants in this library that used 4-MUP was 30.68 (Figure 1G, orange peak; compare also to Figure 2a of reference (29)), suggesting that deoxyribozymes that use CDP-Star and 4-MUP form different structures. Analysis of individual sequences from the evolved library provided additional support for this idea: most contained mutations that were not consistent with the sequence requirements of Supernova (Supplementary Figure S2). The substrate specificities of these new deoxyribozymes also differed from that of Supernova. For example, the deoxyribozyme Aurora 1 could use 4-MUP but not CDP-Star as a substrate, whereas Supernova reacted efficiently with CDP-Star but not 4-MUP (Figure 1H). Similar results were obtained in a complementary study in which we selected for library members that react with the colorimetric substrate pNPP (19). These results demonstrate that our method can be used to identify new fluorescent deoxyribozymes. They also suggest that, despite being isolated from a library based on Supernova, most variants in this library that use 4-MUP as a substrate form structures that are distinct.

**Table 1.** Fluorescent properties of 4-MU, the fluorescent product generated by Aurora. Values were measured at pH 10 (27,28)

Property	Value
Maximum excitation wavelength (λ_max_)	360 nm
Maximum emission wavelength (λ_em_)	450 nm
Extinction coefficient (ϵ)	17 000 M^−1^ cm^−1^
Quantum yield (Φ)	0.63
Brightness (ϵ × Φ)	10 710 M^−1^ cm^−1^

### The catalytic core of Aurora is a 47-nucleotide bulged hairpin

We chose the fluorescent deoxyribozyme from the initial selection with the highest activity for further characterization (Figure 2A). We named this sequence Aurora 1 full-length, and the catalytic motif encoded by this sequence Aurora. One goal was to characterize the secondary structure, sequence requirements, and minimized catalytic core of Aurora. Another was to identify variants with improved catalytic efficiencies. To address both of these goals, we synthesized a second library by randomly mutating the sequence of Aurora 1 full-length at a rate of 21% per position (29–31). At this rate of mutagenesis, all possible variants within about four mutations of the starting sequence were expected to be present in the library (Supplementary Table S2) (32). Catalytically active variants were then identified by artificial evolution and characterized by high-throughput sequencing (Supplementary Figure S3 and Supplementary Tables S3–S5). Initial analysis of these sequences revealed two highly conserved regions (corresponding to nucleotides 1–10 and 43–79) separated by 32 less conserved positions (Figure 2B). The catalytic activity of a 47 nucleotide deoxyribozyme in which the nucleotides at positions 11–42, 80–85, and in the 3′ primer binding site were deleted (called Aurora 1) was similar to that of the full-length sequence (Supplementary Figure S4). The proton NMR spectrum of the 17C 40G mutant of Aurora 2 suggests that Aurora forms a structure containing multiple Watson–Crick base pairs (Figure 2C). Consistent with this observation, comparative sequence analysis (31,33,34) revealed four pairs of covarying positions in the deoxyribozyme (positions 11 and 47, 12 and 46, 17 and 40, and 26 and 30) with mutational patterns consistent with those expected of base pairs. Mutagenesis experiments in which these putative base pairs were disrupted by point mutations and restored by compensatory mutations generally provided strong additional support for the proposed interactions (Figure 2D and Supplementary Figure S5). When interpretated in the context of the conserved nucleotides that flank these base pairs, the 11–47, 12–46, 17–40 and 26–30 constraints suggest that Aurora forms an 11-base pair hairpin interrupted by an asymmetric bulge (Figure 2E). This hairpin contains two irregular features: a TT mismatch (which could reflect a noncanonical interaction) and a highly conserved bulged guanine. The hairpin is capped by a three-nucleotide loop formed by positions 27, 28 and 29. One of the most highly enriched mutations identified in the selection (29 G to A) occurred in this loop (Supplementary Figure S3). In addition, several correlations identified by mutual information analysis (including 22–29, 22–27, 23–26, 26–29 and 22–30; Figure 2E-F and Supplementary Figure S6) suggest that this loop interacts with the conserved asymmetric bulge formed by positions 20–23 and 33–37 rather than extending into solution. Covariation analysis suggests that nucleotides at the 5′ end of Aurora (which include the phosphorylation site) do not form canonical base pairs with one another or with the rest of the deoxyribozyme. However, a network of correlations among positions 4, 6 and 45 (including an AT to GA covariation between positions 4 and 45 which is one of the strongest in the dataset) is consistent with a tertiary interaction that anchors the 5′ end of Aurora to the rest of the catalytic core. It is possible that this interaction helps to position the nucleophilic 5′ hydroxyl group in the vicinity of the phosphate group of 4-MUP. Several additional correlations (including the 16–34, 6–10, 6–46, 6–12, 6–9, 16–37 and 6–42 pairs; Supplementary Figure S6) are consistent with the idea that the 5′ end of Aurora is also facing the asymmetric bulge. If this is the case, the overall architecture of Aurora is likely a bent hairpin in which nucleotides distant in both the primary sequence and secondary structure converge on this bulge.

### Aurora generates a robust fluorescent signal

To evaluate the extent to which these artificial evolution experiments yielded improved variants of Aurora, we compared the catalytic activity of the initial isolate (Aurora 1; Figure 2E) with that of the variant with the highest read number from the randomly mutagenized library (Aurora 2; Figure 2F) (see also Figure 2A for more information about the evolutionary lineage of these deoxyribozymes). Each variant was characterized in the context of the 47-nucleotide minimized catalytic core, and measurements were performed over a range of 4-MUP concentrations using a ligation assay (which measures the extent of self-phosphorylation). These experiments revealed that the catalytic activity of Aurora 2 was more than 100-fold higher than that of Aurora 1 at some substrate concentrations (Figure 2G). Most mutations in Aurora 2 occurred in either the asymmetric bulge (positions 20–23 and 33–37) or the loop (positions 27–29; Figure 2F), highlighting the importance of these parts of the deoxyribozyme. Surprisingly, 4-MUP concentration affected activity in a slightly cooperative way, and evidence for cooperativity was also observed in proton NMR experiments in which Aurora folding was characterized as a function of 4-MUP concentration (Supplementary Figure S7). This could indicate that Aurora contains multiple binding sites for 4-MUP, or a single site that binds multiple 4-MUP molecules. At saturating substrate concentrations, the rate of Aurora 2 of 0.18 min^−1^ (Figure 2G) was similar to the *k*_cat_ of Supernova (20) of 0.15 min^−1^. The concentration of substrate at which activity was half maximal (30 μM for Aurora and 150 μM for Supernova) was also comparable for these two deoxyribozymes.

**Figure 3.**
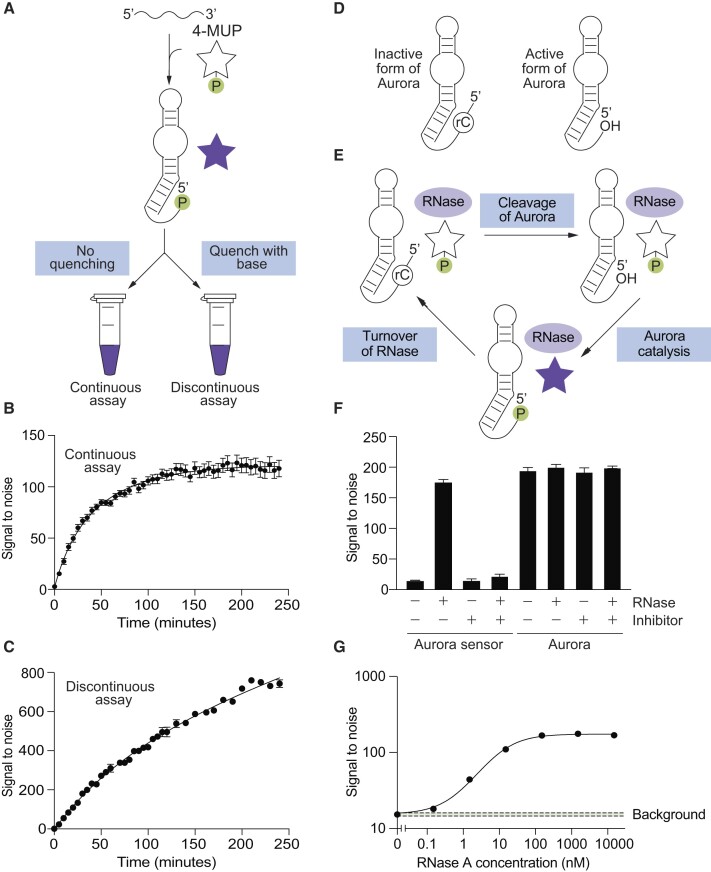
Aurora generates a robust fluorescent signal. (**A**) Workflow of continuous and discontinuous assays using Aurora. (**B**) Example of a continuous assay in which the reaction is continually monitored in a plate reader. (**C**) Example of a discontinuous assay in which time points are quenched with base before measuring fluorescence. (**D**) Design of a ribonuclease sensor based on Aurora that is activated by RNA cleavage. (**E**) Amplification of the single-turnover signal generated by Aurora in the presence of a ribonuclease that promotes a multiple turnover reaction. (**F**) An Aurora sensor with the architecture shown in panel D is activated by RNase A, but not when a ribonuclease inhibitor is present. In contrast, the catalytic activity of Aurora itself is not affected by either RNase A or this ribonuclease inhibitor. (**G**) The Aurora sensor detects ribonuclease A with a limit of detection of 100 pM. Reactions were incubated for 4 h in the presence of the indicated concentration of RNase A, and after quenching with base, fluorescence was measured using a plate reader. The green box indicates the average plus or minus three standard deviations of the background signal to noise ratio measured in the absence of RNase A. See Supplementary Figure S23 for more information about the detection limit of the sensor. Experiments shown in panels B and C were performed using Aurora 2, which those in panels F and G were performed using the sensor shown in Supplementary Figure S22.

In a complementary series of experiments, we investigated the extent to which Aurora enhances fluorescence. When using an experimental setup in which 4-MUP and buffer was mixed with Aurora 2 and fluorescence was continuously monitored using a plate reader (Figure 3A), signal to noise ratios of 10-fold were obtained in minutes and 100-fold in hours (Figure 3B). A stable signal was observed over the course of this experiment, indicating that the product is relatively stable under these conditions. When using a discontinuous setup in which reactions were quenched with base before measurement (which can enhance fluorescence and also increase the stability of the fluorescent product (35)), signal to noise ratios were about 6-fold higher, and values exceeding 700-fold could be achieved (Figure 3C). In both assays, the fluorescent signal generated by Aurora 2 was at least 10-fold higher than that of Aurora 1 (Supplementary Figure S8). Maximum signal to noise ratios in both the absence of base (continuous assay) and the presence of base (discontinuous assay) were similar to those obtained from samples containing synthetic 4-MU at the same concentration as that of 4-MUP used in our assays (Supplementary Figure S9). This indicates that Aurora generates the maximum possible signal to noise ratio for 4-MUP in solution, although it is possible that higher signal to noise ratios could be achieved by deoxyribozymes that enhance the fluorescence of 4-MU when it is bound to the deoxyribozyme (3,4,12–14).

### Aurora requires multiple zinc ions for structure and function

Although our selection experiments provided extensive information about the sequence requirements of Aurora, they revealed little about how external factors (such as components of the buffer) influence the reaction. Such factors can significantly affect signal to noise ratios, and can also provide clues about catalytic mechanisms. We were especially interested in the effects of metal ions on the reaction because they can play both structural and catalytic roles in ribozymes and deoxyribozymes (36,37). Our survey of reaction conditions revealed both differences and similarities between Aurora and Supernova (Supplementary Figures S10-S20) (20,38) as well as between Aurora and the colorimetric deoxyribozyme Apollon (19). An important difference was that Aurora appears to require monovalent ions for activity (Supplementary Figures S10-S12) while Supernova (20,38) and Apollon (19) do not. On the other hand, Aurora (Supplementary Figure S13-S14), Supernova (20,38) and Apollon (19) each require zinc. The dependence of catalytic rate on zinc concentration is also highly cooperative for these three deoxyribozymes (Supplementary Figure S14, (38,19), suggesting that multiple zinc ions are needed for function. This is intriguing because zinc ions play catalytic roles in some protein enzymes (such as alkaline phosphatase (39,40)) that catalyze reactions similar to that promoted by Aurora. To determine whether these metal ion requirements in part reflect structural roles, proton NMR was used to directly monitor the effects of different metal ions on deoxyribozyme folding. The results of these experiments were similar to those that used catalytic activity as a readout. For example, chemical shifts consistent with canonical base pairs were observed in a buffer that contained zinc and potassium, but not in buffers that lacked either zinc or potassium (Supplementary Figure S10). NMR experiments also provided additional evidence that zinc affects Aurora folding in a highly cooperative way (Supplementary Figure S20). Zinc also plays an important role in deoxyribozymes that cleave DNA (41–43), suggesting a more general role for this ion in the context of nucleic acid enzymes that promote phosphoryl transfer reactions (44). Taken together, these experiments indicate that both zinc and a monovalent metal ion (but not necessarily potassium) are needed for Aurora folding and function. They also highlight possible mechanistic similarities among the chemiluminescent, fluorescent, and colorimetric deoxyribozymes recently identified in our group.

### Engineered forms of Aurora can detect ligands and enzymes in solution

Variants of Aurora that only generate fluorescence in the presence of an input of interest could be useful for applications such as high-throughput screening and diagnostics. This is especially true for variants that can be activated in solution without the need for wash steps or biochemical purifications. To determine whether the catalytic activity of Aurora can be modulated by ligands, we used rational design to construct a programmable sensor that only produces fluorescence in the presence of specific oligonucleotide sequences (Supplementary Figure S21). This sensor produced significantly more fluorescence in the presence of the target than in its absence, could be programmed to detect a range of targets, and was only activated by oligonucleotides that it was designed to detect (Supplementary Figure S21). However, its sensitivity was low, with a limit of detection of approximately 1 μM of target (Supplementary Figure S21). This is likely related to catalytic turnover because, unlike classical enzymes, a single molecule of Aurora can only generate one molecule of fluorescent product.

To improve sensitivity, we investigated whether it was possible to link the single-turnover signal generated by Aurora to the catalytic activity of an enzyme that itself catalyzes a multiple turnover reaction. Because Aurora is made of DNA, we expected that this type of coupling would be easiest to achieve using enzymes that modify nucleic acids, and set out to develop a variant of Aurora that is activated by enzymes that cleave RNA. Our sensor was constructed by fusing a short DNA oligonucleotide containing a ribonucleotide at its 3′ end to the 5′ end of Aurora (Figure 3d and Supplementary Figure S22). Because Aurora uses its 5′ hydroxyl group as the nucleophile in the reaction, this modification was expected to abolish catalytic activity and eliminate the production of fluorescence. In the presence of a ribonuclease that cleaves RNA at internal sites to generate 3′ phosphate (or 2′-3′ cyclic phosphate) and 5′ hydroxyl termini, however, the RNA linkage should be cleaved, which will regenerate the 5′ end of Aurora and restore catalytic activity (Figure 3D and Supplementary Figure S22). Because protein ribonucleases are generally capable of multiple turnover catalysis, this architecture was also expected to amplify the single-turnover signal generated by Aurora (Figure 3E). We tested our sensor using ribonuclease A. This activated the sensor and enhanced fluorescence more than 10-fold (Figure 3F). Furthermore, the detection limit of the sensor under these conditions (∼100 pM; defined here as the minimum concentration of RNase A that gives a signal ≥ 3 standard deviations higher than that of the average background value measured in the absence of RNase A) was approximately 10 000-fold lower than our oligonucleotide sensor (compare Figures 3G and Supplementary Figure S21, and see also Supplementary Figure S23). This dramatic increase in sensitivity is likely due to the high turnover number of RNase A. To further probe the mechanism of this sensor, we investigated whether activation was affected by RiboLock, a commercially available inhibitor of RNase A. RiboLock had no effect on Aurora itself (Figure 3F, right), but prevented the Aurora sensor from being activated by RNase A (Figure 3F, left). This provided additional evidence that the sensor is activated by RNA cleavage. Taken together, these experiments show that assays which use a covalently blocked form of Aurora to detect a multiple-turnover enzyme can be orders of magnitude more sensitive than those that use unmodified Aurora. They also indicate that such a sensor can be used to detect the presence of ribonuclease inhibitors in a sample.

### Using Aurora to identify Nsp15 inhibitors in a high-throughput screen

**Figure 4.**
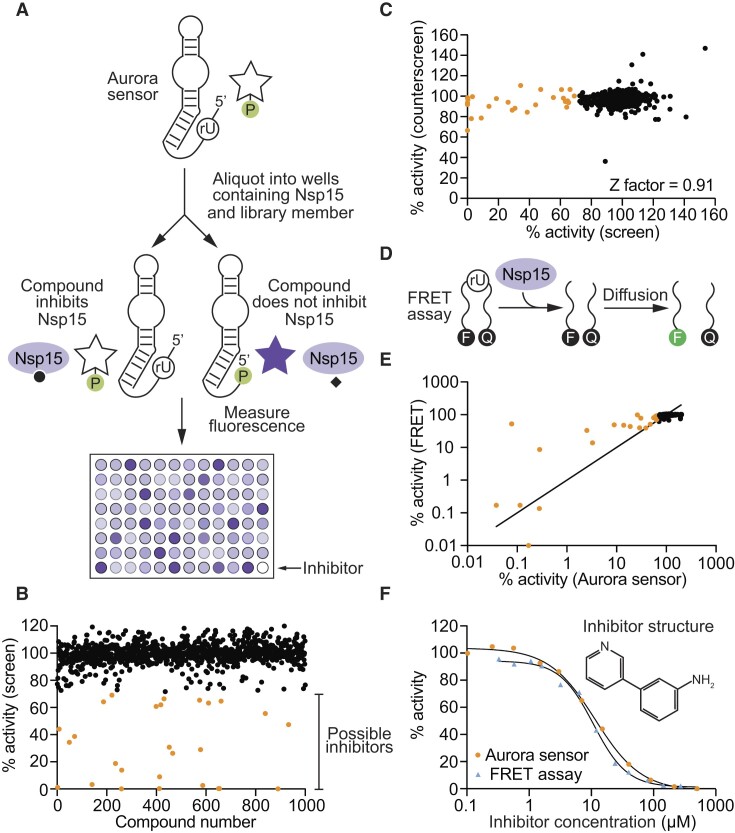
Identification of small-molecule inhibitors of the SARS-CoV-2 ribonuclease Nsp15 using a fluorescent Aurora sensor. (**A**) Workflow of high-throughput screen to identify Nsp15 inhibitors. (**B**) Effect of each compound in the 1000-member library on the fluorescence of the Aurora sensor. Potential inhibitors are shown in orange. (**C**) Identification of inhibitors and false positives. The *x*-axis of the graph shows the fluorescent signal generated by the Aurora sensor in the presence of Nsp15 and different compounds in the library, while the *y*-axis shows the fluorescent signal generated by Aurora itself in the presence of Nsp15 and the same compounds. Points with high fluorescence values on both the *x*-axis and the *y*-axis (shown in black) correspond to wells containing compounds that inhibit neither Nsp15 nor Aurora. Points with low fluorescence values on the *x*-axis and a high fluorescence value on the *y*-axis (shown in orange) correspond to wells containing compounds that inhibit Nsp15 but not Aurora. (**D**) Workflow of a FRET assay for ribonuclease activity. (**E**) Comparison of the results of a high-throughput screen for Nsp15 inhibitors using the Aurora sensor (*x*-axis) with a screen of the same library using a FRET assay (*y*-axis). (**F**) Example of an Nsp15 inhibitor identified in the screen. This compound inhibits Nsp15 with an IC50 value of 12 μM when measured using the Aurora sensor and 11 μM when measured using the FRET assay.

Because assays using Aurora sensors can be performed rapidly and inexpensively, they appear to be well-suited for applications such as high-throughput screens. To further investigate this idea, we investigated whether our Aurora sensor could be used to identify inhibitors of the SARS-CoV-2 endoribonuclease Nsp15. This ribonuclease cleaves 3′ of pyrimidines (preferentially uridines) to generate 2′-3′ cyclic phosphate and 5′ hydroxyl termini (45). It helps to prevent host recognition by degrading double-stranded viral intermediates (45), and inhibitors could potentially be useful as antiviral agents (46). Pilot experiments showed that, as was the case for RNase A, it was possible to construct a version of Aurora that was activated by Nsp15 (Supplementary Figures S22 and S24). To perform a screen using this sensor, a master mix containing Nsp15 and buffer was aliquoted into the wells of 384 well plates, each of which contained a different compound from a 1000-member fragment-based small molecule library (Figure 4A). A second master mix containing the Aurora sensor was then added to each well. After a short incubation to allow Nsp15 to cleave the RNA linkage and activate the sensor, zinc and 4-MUP were added to initiate deoxyribozyme catalysis. After another incubation, fluorescence was measured in a plate reader (Figure 4B). In wells containing compounds that do not inhibit Nsp15, cleavage of the RNA linkage in the Aurora sensor by Nsp15 was expected to activate the sensor and lead to production of a fluorescent signal (Figure 4B, black points). In contrast, RNA cleavage should not occur and fluorescence should not be produced in wells containing compounds that inhibit either Nsp15 or Aurora itself (Figure 4B, orange points). To distinguish compounds that inhibit Nsp15 from those that inhibit Aurora, a counterscreen was performed using Aurora rather than the Aurora sensor. A graph comparing these two screens revealed that none of the hits identified in the initial screen inhibited Aurora in the counterscreen (Figure 4C). This indicates that these hits are Nsp15 rather than deoxyribozyme inhibitors, and also that they do not quench fluorescence of 4-MU itself. To compare these results to those obtained using standard methods, the screen was repeated using a FRET assay in which Nsp15 was incubated with library members and a DNA substrate containing a fluorophore at one end and a quencher at the other (Figure 4D). Cleavage by Nsp15 was expected to result in an increase in fluorescence, while the fluorescence in wells containing Nsp15 inhibitors was expected to remain at background levels. The results of this FRET screen were virtually identical to those obtained using the Aurora sensor (Figure 4E). Several hits were further characterized as a function of concentration using both the Aurora sensor and the FRET assay. The most potent of these compounds inhibited Nsp15 with an IC50 of 12 μM in an assay that used the Aurora sensor and 11 μM in an assay that used a FRET assay (Figure 4F). Other hits inhibited Nsp15 with IC50 values ranging from 7.9 to 121 μM (Supplementary Figure S25). These experiments indicate that Aurora sensors can be used in combination with small-molecule libraries to rapidly identify inhibitors in high-throughput screens.

## Conclusions

In this study, we developed a new way to generate fluorescence using a deoxyribozyme called Aurora and a coumarin substrate called 4-MUP. Our approach offers a number of advantages when compared to other methods of generating fluorescent signals. Both Aurora and 4-MUP are stable, inexpensive and widely available. The workflow is simple, and formation of the fluorescent product can be monitored in solution and in real time without the need for wash steps or biochemical purifications. The signal to noise ratio of the fluorescent signal is also higher than that produced using widely used methods like molecular probes. A second goal of this study was to establish that these deoxyribozymes can be used for real-world applications. As an initial proof of concept for this idea, we showed that Aurora can be readily converted by rational design into a sensor that only generates fluorescence in the presence of an input. Our most sensitive sensor could detect ribonucleases with a limit of detection of approximately 100 pM, which compares favorably with the detection limits of many homogenous assays that use aptamers in combination with more expensive signaling elements such as fluorophores, dyes, quantum dots, or gold nanoparticles (47–50). Although selection was not used to optimize this sensor, it could in principle be utilized to improve its performance or to develop sensors that detect other target molecules (9,51–53). After verifying that this sensor worked, it was used to identify inhibitors of the Nsp15 ribonuclease from SARS-CoV-2 in a high-throughput screen. Our assay could readily distinguish between reactions that contained active ribonuclease and those that did not (*Z*-factor = 0.91). It did not produce false positives from compounds that inhibit Aurora rather than Nsp15, although we note that the frequency of such false positives will depend on the properties of the library. It also yielded results that were similar to those obtained when the library was screened in parallel using a more standard FRET assay (54,55). While our assay is comparable to those which use FRET in terms of both simplicity and workflow, reagent costs are several fold lower and signal to noise ratios are considerably higher. We anticipate that further optimization of Aurora using methods such as recombination (56) and secondary structure libraries (57) in combination with single-step (58) and conventional selections will continue to decrease costs and increase signal to noise ratios, which will in turn increase the utility of Aurora for applications such as high-throughput screening and diagnostics. In a more general sense, our work highlights the potential of functional DNA molecules as widely applicable fluorescent tools.

## Supplementary Material

gkae467_Supplemental_File

## Data Availability

The data underlying this article will be shared on reasonable request to the corresponding author.
