# Supplementary material for: Aurora: a fluorescent deoxyribozyme for high-throughput screening
Source: Nucleic Acids Res. 2024 Jun 11;52(15):9049–61. doi: 10.1093/nar/gkae467 (PMC11347150; doi:10.1093/nar/gkae467)
Supplement: gkae467_Supplemental_File [file gkae467_supplemental_file.pdf]

Aurora: a fluorescent deoxyribozyme for high-throughput screening

Martin Volek[1,2], Jaroslav Kurfürst[1,3], Matúš Drexler[1], Michal Svoboda[1], Pavel Srb[1], Václav Veverka[1,4], and Edward A. Curtis[1]*

[1]Institute of Organic Chemistry and Biochemistry of the Czech Academy of Sciences, Prague 166 10, Czech Republic
[2]Department of Genetics and Microbiology, Faculty of Science, Charles University in Prague, Prague 128 44, Czech Republic
[3]Department of Informatics and Chemistry, University of Chemistry and Technology, Prague 166 28, Czech Republic
[4]Department of Cell Biology, Faculty of Science, Charles University in Prague, Prague 128 44, Czech Republic

Tel: +420 733 169 654; Email: curtis@uochb.cas.cz

ABSTRACT: Fluorescence facilitates the detection, visualization, and tracking of molecules with high sensitivity and specificity. A functional DNA molecule that generates a robust fluorescent signal would offer significant advantages for many applications compared to intrinsically fluorescent proteins, which are expensive and labor intensive to synthesize, and fluorescent RNA aptamers, which are unstable under most conditions. Here we describe a novel deoxyriboyzme that rapidly and efficiently generates a stable fluorescent product using a readily available coumarin substrate. An engineered version can detect picomolar concentrations of ribonucleases in a simple homogeneous assay, and was used to rapidly identify novel inhibitors of the SARS-CoV-2 ribonuclease Nsp15 in a high-throughput screen. Our work adds an important new component to the toolkit of functional DNA parts, and also demonstrates how catalytic DNA motifs can be used to solve real-world problems.

SUPPLEMENTARY TABLES

Supplementary Table 1**.** Sequences of deoxyribozymes and oligonucleotides used in this study.

**Name**	**Nucleotide sequence (5' to 3')**
Pool1	GGAAGAGATGGCGACGACACAGGGACGATGCCGAATATCCTCAGTGCGCAGGGCCGCAGGGGGGAGTGACTTGGGATGGGGGGTCCACTAATGATCTGCCCGATG (underlined NTs were mutagenized at the rate of 21%)
FWD1	ACCGCTCAGGTGTAGTATCA
REV1	CATCGGGCAGATCATTAGTG
Splint1	GTCGCCATCTCTTCCTGATACTACACCTGAGCGGT
FWD1r	ACCGCTCAGGTGTAGTATCrA
REV1p	pCATCGGGCAGATCATTAGTG
Aurora 1 full-length	GGAAGAGATGACCAGGGCAGCGGGACGCTGACGAATTTTCTCACTATGTCCGGGACCCGAGGGGCGTGAGGAGTGTTGTGCAATT
Aurora 1	GGAAGAGATGACTATGTCCGGGACCCGAGGGGCGTGAGGAGTGTTGT
Pool2	GGAAGAGATGACCAGGGCAGCGGGACGCTGACGAATTTTCTCACTATGTCCGGGACCCGAGGGGCGTGAGGAGTGTTGTGCAATTCTCATGAACTATCCGCTGGA (underlined NTs were mutagenized at the rate of 21%)
REV2	TCCAGCGGATAGTTCATGAG
REV2p	pTCCAGCGGATAGTTCATGAG
Aurora 2 full-length	GGAAGGGATGAGCAGAGTAGCGCGACGATGACGAATTTTGTAACTATGTCCGGTTCCTGTAAGGCATGTGGAGTGTTGTGCAATT
Aurora 2	GGAAGGGATGACTATGTCCGGTTCCTGTAAGGCATGTGGAGTGTTGT
Aurora 2 17C40G	GGAAGGGATGACTATGCCCGGTTCCTGTAAGGCATGTGGGGTGTTGT
Oligo sensor 1	GGAAGGGATGGAAGGTCAATACTATGTCCGGTTCCTGTAAGGCATGTGGAGTGTTGTATTGACCTTCATTGACCTTC
Oligo sensor 2	GGAAGGGATGGGGCACTGATACTATGTCCGGTTCCTGTAAGGCATGTGGAGTGTTGTATCAGTGCCCAGTGGGCACG
Oligo sensor 3	GGAAGGGATGGATGATCGGAACTATGTCCGGTTCCTGTAAGGCATGTGGAGTGTTGTTCCGATCATCCGAAGATCAG
Oligo sensor 4	GGAAGGGATGAAGTAATAGCACTATGTCCGGTTCCTGTAAGGCATGTGGAGTGTTGTGCTATTACTTATCTTTCCGA
Oligo sensor 5	GGAAGGGATGTAAAAGATAAACTATGTCCGGTTCCTGTAAGGCATGTGGAGTGTTGTTTATCTTTTATTCGTGTGTA
Target Oligo 1	GAAGGTCAATGAAGGTCAAT
Target Oligo 2	CGTGCCCACTGGGCACTGAT
Target Oligo 3	CTGATCTTCGGATGATCGGA
Target Oligo 4	TCGGAAAGATAAGTAATAGC
Target Oligo 5	TACACACGAATAAAAGATAA
RNase A sensor	AAAArCGGAAGGGATGAATATGTCCGGTTCCTTTTAGGCGTGTGGAGTGTTTT
Nsp15 sensor	AAAArUGGAAGGGATGAATATGTCCGGTTCCTTTTAGGCGTGTGGAGTGTTTT

Supplementary Table 2. Coverage of sequence space in a randomly mutagenized library. This example shows expected coverage in a library generated by randomly mutagenizing 85 positions in the full-length version of Aurora at a rate of 21% per position. The "probability" column indicates the probability that a sequence generated during the synthesis will contain the indicated number of mutations relative to the starting sequence. The "possible" column indicates the total number of possible sequences with the indicated number of mutations relative to the starting sequence. The "average copies" column was calculated by multiplying the value in the "probability" column by 10^{14} (the number of sequences in the library) and dividing by the value in the "possible" column. See Knight and Yarus (2003) for more information about these types of calculations.

Mutations	Probability	Possible	Average copies
0	1.99×10^{-9}	1	1.99×10^{5}
1	4.49×10^{-8}	2.55×10^{2}	1.76×10^{4}
2	5.01×10^{-7}	3.21×10^{4}	1.56×10^{3}
3	3.69×10^{-6}	2.67×10^{6}	1.38×10^{2}
4	2.01×10^{-5}	1.64×10^{8}	1.22×10^{1}
5	8.65×10^{-5}	7.97×10^{9}	1.09×10^{0}
6	3.07×10^{-4}	3.19×10^{11}	9.61×10^{-2}
7	9.20×10^{-4}	1.08×10^{13}	8.52×10^{-3}
8	2.38×10^{-3}	3.16×10^{14}	7.55×10^{-4}
9	5.42×10^{-3}	8.01×10^{15}	6.69×10^{-5}
10	1.10×10^{-2}	1.85×10^{17}	5.93×10^{-6}
11	1.99×10^{-2}	3.78×10^{18}	5.25×10^{-7}
12	3.26×10^{-2}	6.99×10^{19}	4.66×10^{-8}
13	4.86×10^{-2}	1.18×10^{21}	4.13×10^{-9}
14	6.64×10^{-2}	1.82×10^{22}	3.66×10^{-10}
15	8.36×10^{-2}	2.58×10^{23}	3.24×10^{-11}

Supplementary Table 3. Number of unique sequences with different read numbers in a library of Aurora variants after selection. This library was generated by randomly mutagenizing 85 positions in the full-length version of Aurora at a rate of 21% per position. After enriching the library for catalytically active variants using artificial evolution, it was characterized by high-throughput sequencing.

**Read number**	**Unique sequences**
≥ 1	849,535
≥ 3	232,121
≥ 10	84,984
≥ 30	32,791
≥ 100	9,856
≥ 300	2,561
≥ 1,000	424
≥ 3,000	50
≥ 10,000	3

Supplementary Table 4. Relationship between mutational frequencies in the evolved library and catalytic activity for mutations in unpaired positions. Mutations were tested in the background of Aurora 2.

Enrichment values from high-throughput sequencing			Percent activity relative to Aurora	
**Original nt**	**Mutation**	**Enrichment**	**Average**	**SD**
29A	29T	9.22	94	4
34A	34T	8.87	103	9
27G	27T	4.48	85	6
23T	23C	4.18	69	4
37T	37C	3.96	82	4
22T	22G	3.82	64	3
28T	28A	3.16	99	3
37T	37A	2.62	94	4
27G	27A	2.53	100	10
23T	23A	1.62	91	9
10G	10A	1.27	103	3
35T	35A	1.27	82	4
27G	27C	1.27	80	5
34A	34G	1.27	95	4
28T	28C	1.27	72	3
6G	6A	1.27	74	1
10G	10T	1.27	95	4
9T	9A	1.27	90	8
29A	29C	1.26	72	7
28T	28G	1.26	85	1
9T	9G	1.25	24	2
34A	34C	1.25	86	2
35T	35C	1.24	80	2
22T	22A	1.23	70	6
8A	8T	1.22	101	5
8A	8G	1.20	89	1
13T	13A	1.19	91	5
6G	6T	1.18	65	7
7G	7A	1.16	93	9
10G	10C	1.13	86	3
9T	9C	0.97	50	6
22T	22C	0.92	93	5
45T	45C	0.82	13	1
45T	45G	0.79	79	4
7G	7T	0.78	96	3
35T	35G	0.74	70	3
29A	29G	0.69	52	9
37T	37G	0.65	15	1
33C	33G	0.62	11	3
13T	13G	0.59	69	3
33C	33A	0.55	13	1
8A	8C	0.53	86	2
23T	23G	0.44	7	1
13T	13C	0.40	27	5
4A	4C	0.39	10	3

Supplementary Table 5. Relationship between mutational frequencies in the evolved library and catalytic activity for mutations in base pairs. Mutations were tested in the background of Aurora 2.

Enrichment values from high-throughput sequencing			Percent activity relative to Aurora	
Original bp	Mutation	Enrichment	Average	SD
4A 45T	4G 45A	7.73	89	4
12C 46G	12A 46T	2.81	81	5
17T 40A	17C 40G	2.45	102	1
26T 30A	26C 30G	1.46	77.5	0.7
16G 42T	16G 42C	1.43	86	2
26T 30A	26T 30G	1.30	20.2	0.3
16G 42T	16A 42T	1.05	0	n.d.
26T 30A	26A 30T	0.77	96	3
11A 47T	11G 42C	0.69	84	2
16G 42T	16A 42C	0.47	1.7	0.2
12C 46G	12G 46C	0.36	56	4
12C 46G	12T 46A	0.24	35	2
11A 47T	11T 47A	0.22	44	2
12C 46G	12G 46T	0.21	60	12
12C 46G	12T 46T	0.20	30	1
18C 39G	18T 39G	0.19	80	11
4A 45T	4A 45A	0.17	111	2
18C 39G	18T 39A	0.14	103	2
26T 30A	26G 30C	0.088	75	4
11A 47T	11C 47G	0.065	43	6
12C 46G	12T 46G	0.038	27	5
11A 47T	11G 47T	0.024	94	4
4A 45T	4G 45C	0.0024	0	n.d.
19C 38G	19T 38A	0.0018	12	1
15T 43G	15C 43G	0.00071	0	n.d.
15T 43G	15T 43A	0.00039	0	n.d.
25C 31G	25T 31A	0.00019	0	n.d.
14A 44T	14C 44G	0.000062	0	n.d.
24C 32G	24T 32T	0	0	n.d.

SUPPLEMENTARY FIGURES

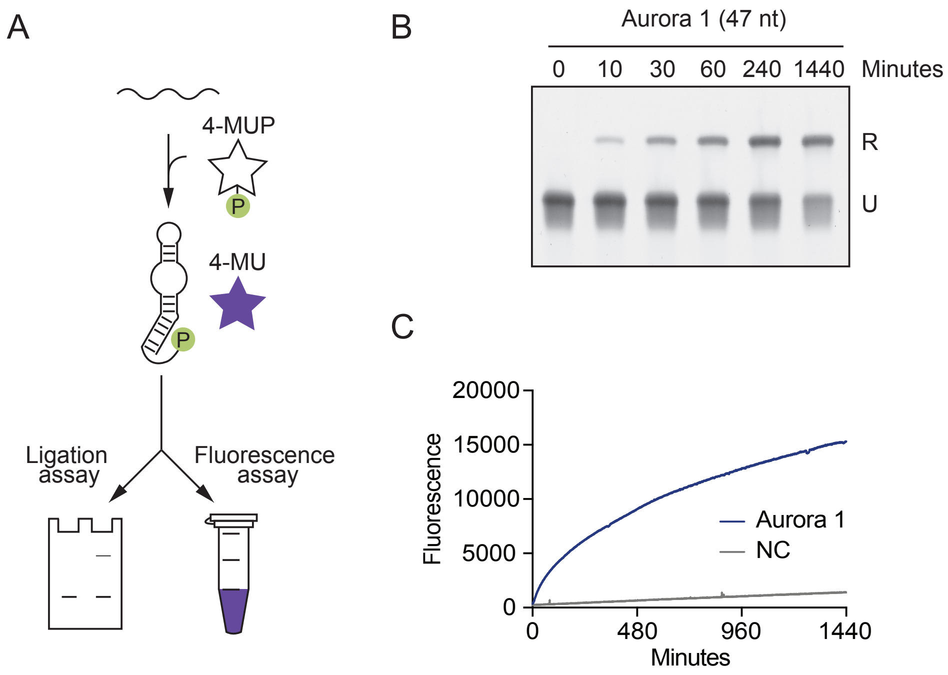

Supplementary Figure 1. Deoxyribozymes identified in the selection can phosphorylate themselves and generate fluorescence in the presence of 4-MUP. (A) Assays for self-phosphorylation and fluorescence. (B) Example of a self-phosphorylation reaction analyzed using the ligation assay. (C) Example of a fluorescence assay analyzed using a plate reader. The initial rate of the deoxyribozyme-catalyzed reaction is 52-fold faster than that of the background reaction under these conditions. Experiments were performed using Aurora 1 (note that the signal to noise ratio of Aurora 2 is 15-fold higher than that of Aurora 1).

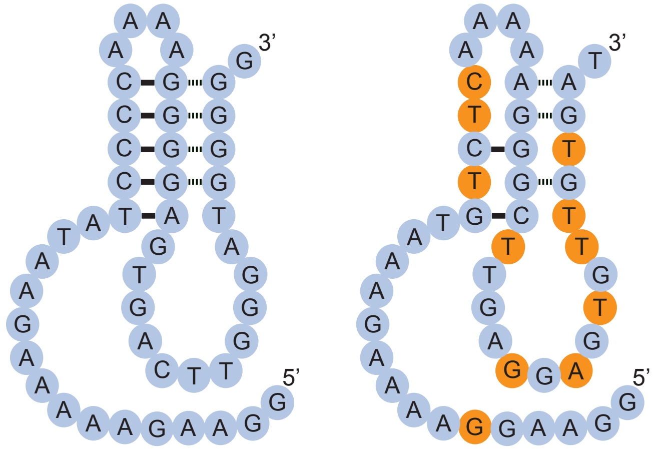

Supplementary Figure 2. Aurora cannot form the secondary structure of Supernova. Left: secondary structure of the catalytic core of Supernova. Right: sequence of the homologous positions in Aurora 1 (corresponding to nucleotides 1-6, 33-42, and 61-82) mapped onto the secondary structure of Supernova. Positions shown in orange disrupt base triples or are otherwise inconsistent with the sequence requirements of Supernova.

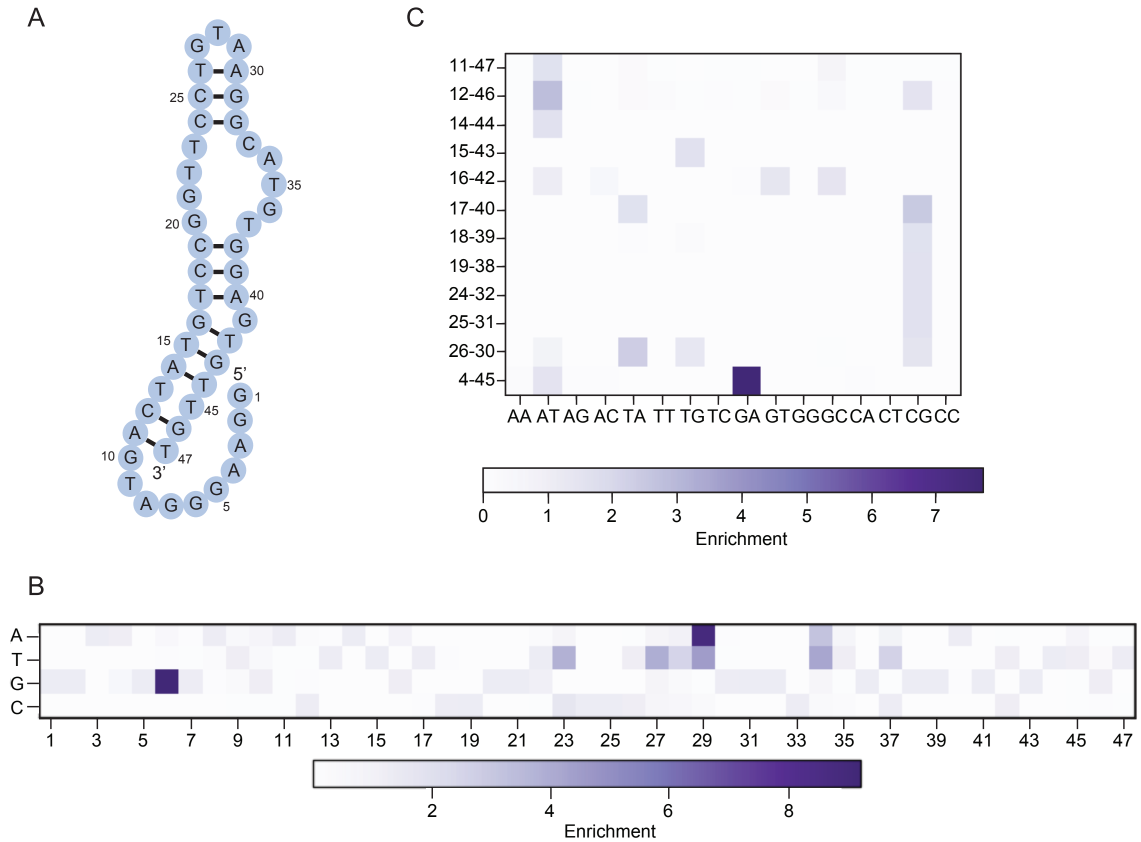

Supplementary Figure 3. Mutations enriched after selection using a randomized mutagenized library. (A) Secondary structure model of Aurora 2. (B) Heat map showing enriched mutations at each position in the minimized catalytic core of Aurora. (C) Heat map showing enriched base pairs in Aurora. Note that virtually all of the enriched sequences correspond to those that can form canonical base pairs (A-T, T-A, C-G or G-C). Enrichment is defined as the frequency of a nucleotide or base pair in the evolved library divided by its frequency in the starting library.

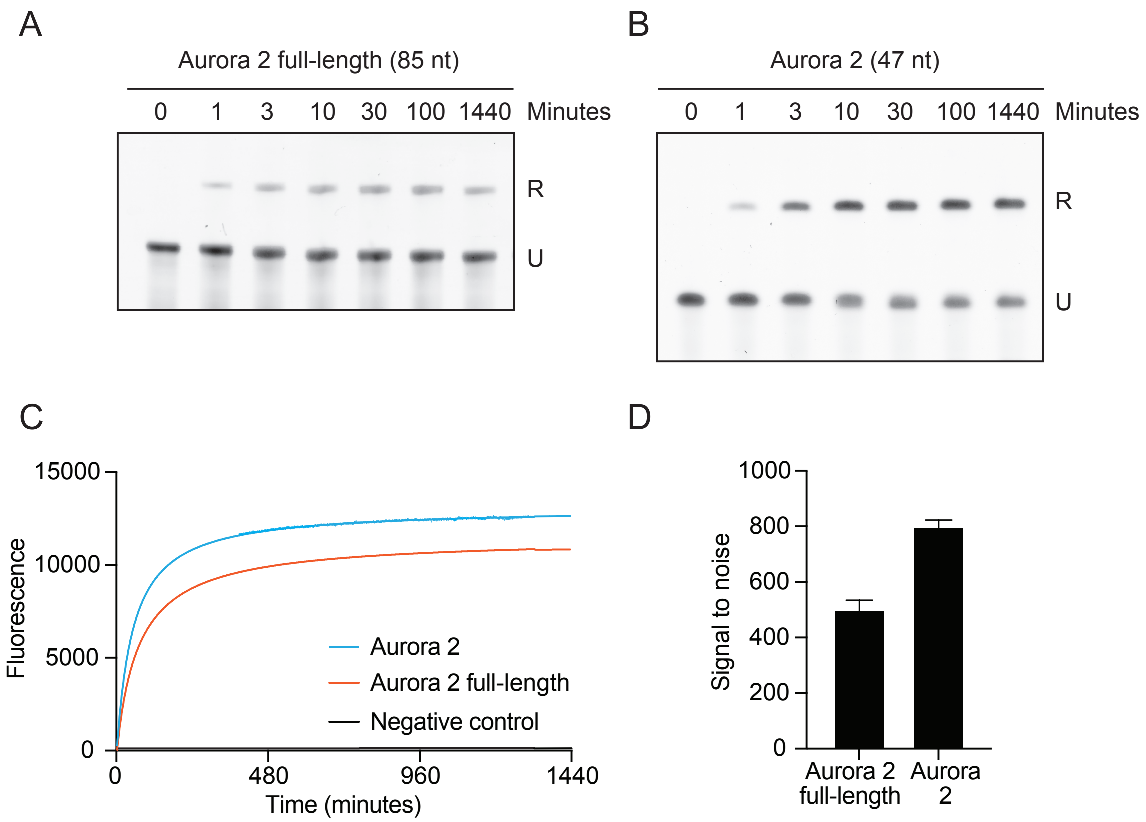

Supplementary Figure 4. Catalytic active of full-length and minimized Aurora. (A) Time course of the 85-nucleotide full-length Aurora 2 deoxyribozyme measured using the ligation assay. Ligations were performed after incubating 1 µM full-length Aurora with 1 mM 4-MUP at the indicated times. (B) Same experiment as in panel A, but for the 47-nucleotide minimized catalytic core of Aurora 2. (C) Fluorescence production of full length and minimized versions of Aurora 2. Reactions were performed using 15 µM DNA and 30 µM 4-MUP in a buffer containing 200 mM KCl, 1 mM $ZnCl_2$, 5% (v/v) DMSO, and 50 mM HEPES, pH 7.4. Fluorescence was measured for 24 hours using a TECAN Spark plate reader. (D) Signal to noise ratio of full-length and minimized versions of Aurora 2. Reactions were performed using 15 µM DNA and 30 µM 4-MUP in a buffer containing 200 mM KCl, 1 mM $ZnCl_2$, 5% (v/v) DMSO, and 50 mM HEPES, pH 7.4. After incubating for 4 hours, 20 µl of 1 M KOH was added and fluorescence was measured using a TECAN Spark plate reader. Signal to noise ratio is defined as the rate of production of fluorescence in the presence of deoxyribozyme divided by the rate in the absence of deoxyribozyme. Columns show the average of three experiments, and error bars represent one standard deviation.

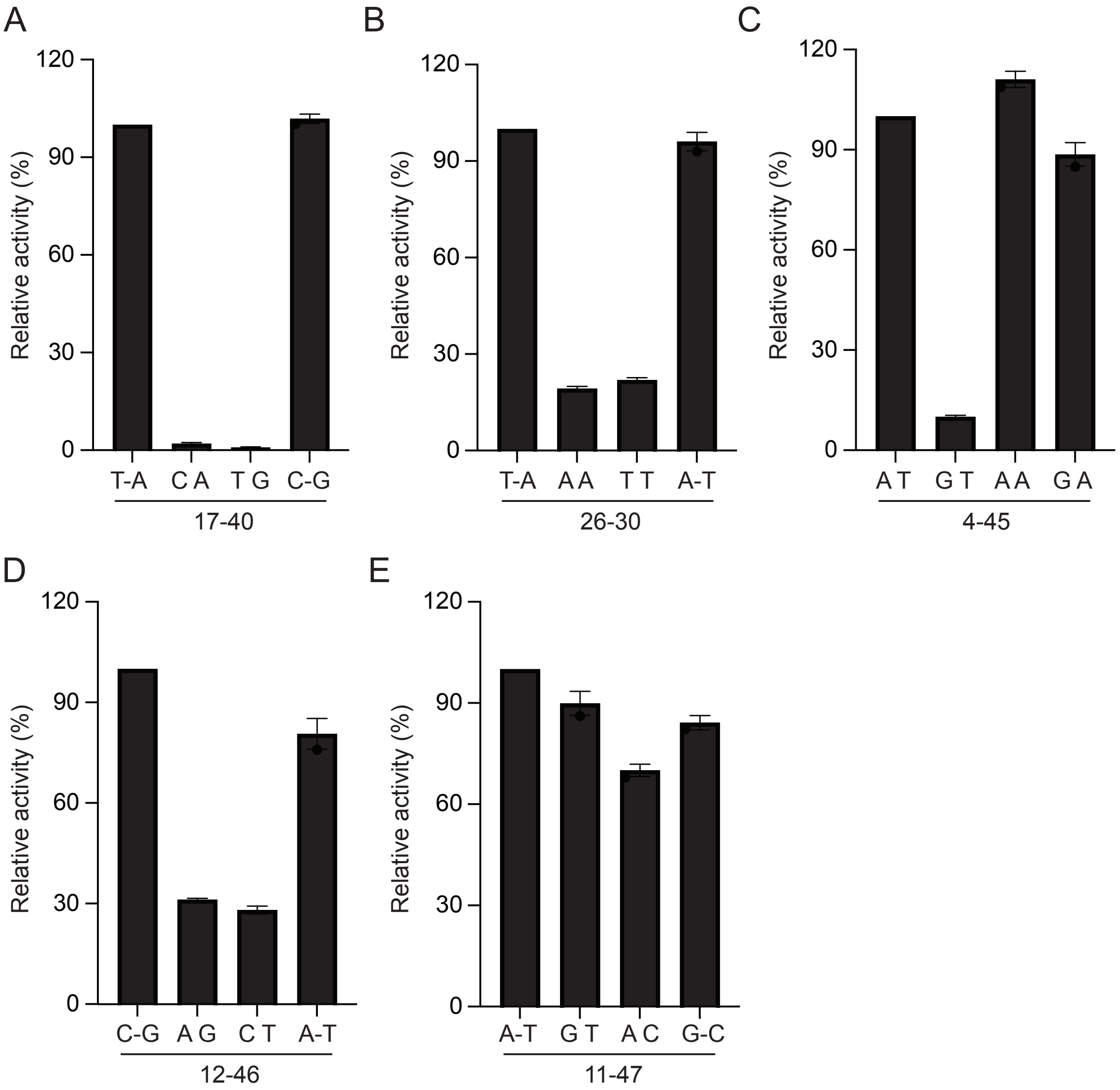

Supplementary Figure 5. Testing base pairs and noncanonical interactions in Aurora using double-mutant cycles. (A) Testing the 17-40 base pair. Signal to noise ratios are expressed relative to a variant containing a T-A base pair. (B) Testing the 26-30 base pair. Signal to noise ratios are expressed relative to a variant containing a T-A base pair. (C) Testing the 4-45 noncanonical pair. Signal to noise ratios are expressed relative to a variant containing an A T pair. (D) Testing the 12-46 base pair. Signal to noise ratios are expressed relative to a variant containing a C-G base pair. (E) Testing the 11-47 base pair. All signal to noise ratios are expressed relative to a variant containing a A-T base pair. Reactions were performed using 15 µM Aurora and 30 µM 4-MUP in a buffer containing 200 mM KCl, 1 mM $ZnCl_2$, 5% (v/v) DMSO, and 50 mM HEPES, pH 7.4. After incubating for 4 hours, 20 µl of 1 M KOH was added and fluorescence was measured using a TECAN Spark plate reader. Signal to noise ratio is defined as the rate of production of fluorescence in the presence of deoxyribozyme divided by the rate in the absence of deoxyribozyme. Columns show the values from three experiments, and error bars represent one standard deviation. Experiments were performed using Aurora 2.

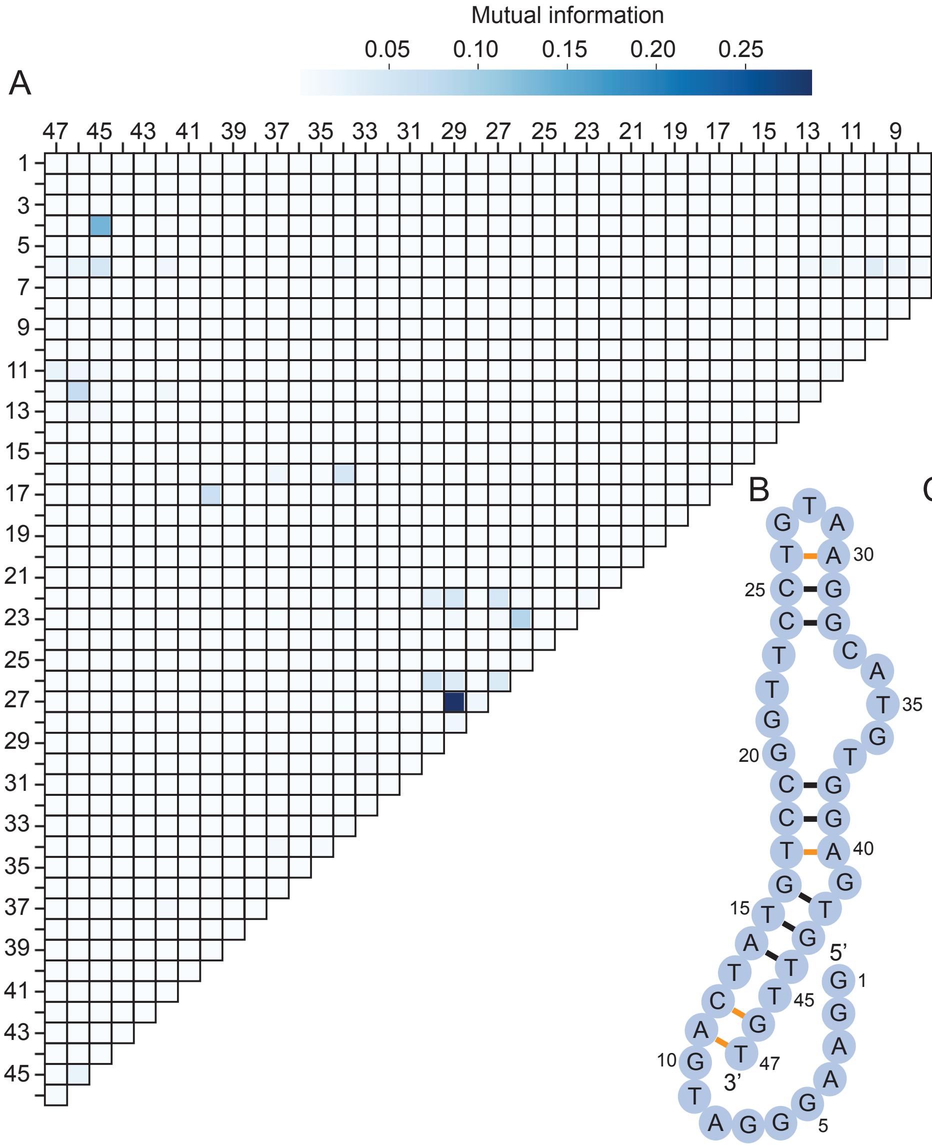

C

Pair	MI
27-29	0.287
4-45	0.136
23-26	0.087
12-46	0.070
17-40	0.063
26-30	0.051
6-45	0.048
16-34	0.047
22-29	0.047
22-27	0.046
26-27	0.040
26-29	0.039
4-6	0.039
6-10	0.035
22-30	0.029
45-46	0.024
11-47	0.024
6-46	0.024
6-12	0.023
6-9	0.020
6-42	0.015
11-46	0.015
27-28	0.014
16-37	0.014
28-29	0.013

Supplementary Figure 6. Identification of correlated pairs of positions in Aurora using mutual information. (A) Heat map showing mutual information correlations between different pairs of positions in the minimized catalytic core of Aurora. (B) Secondary structure model of Aurora. Base pairs are shown using solid black lines, and base pairs supported by covariation analysis are shown in orange. (C) Mutual information values of the 25 pairs of positions with the highest mutual information values. Pairs of positions that form base pairs are shown in orange.

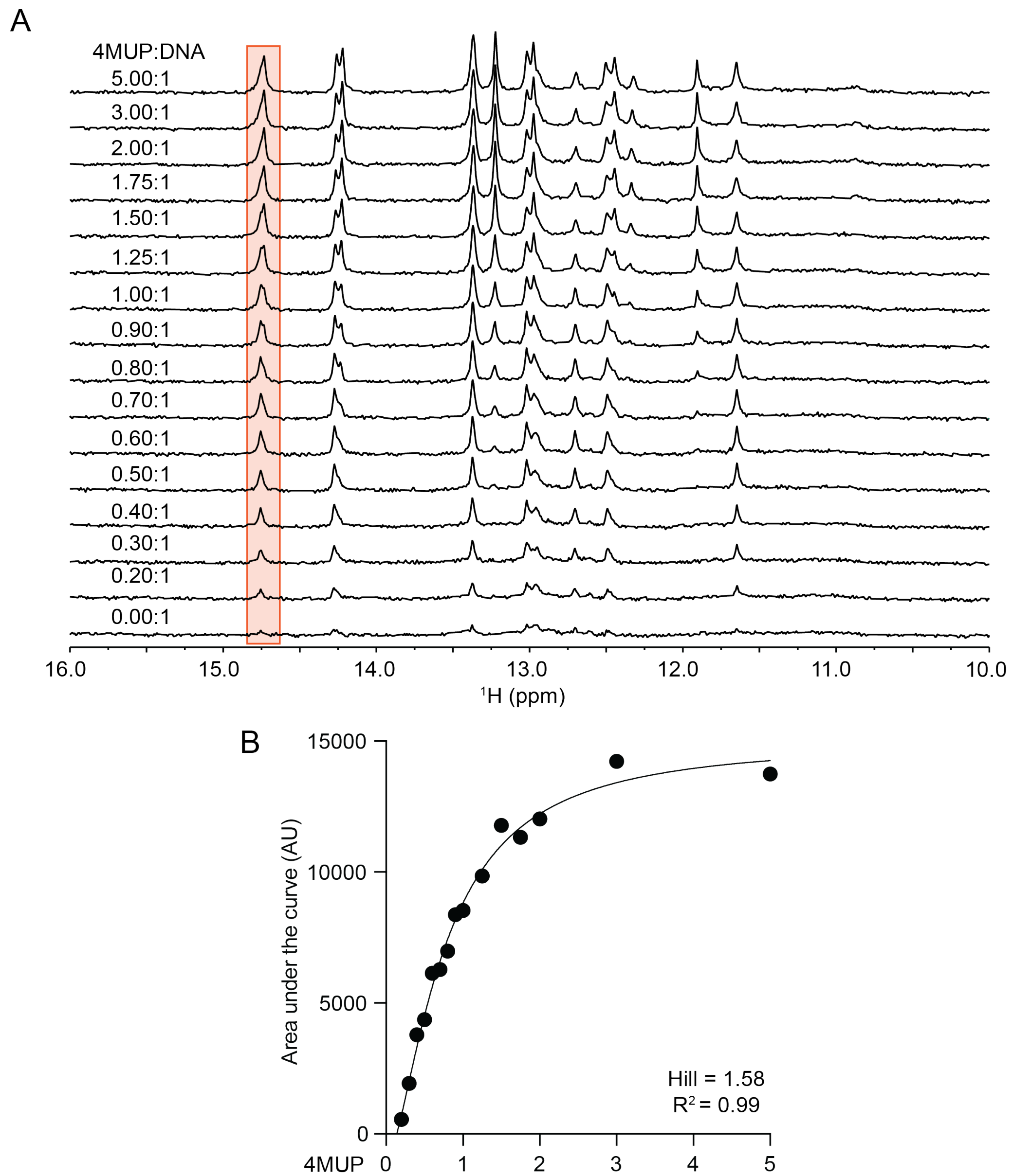

Supplementary Figure 7. Cooperative effect of 4-MUP on Aurora folding. (A) Proton NMR spectra of the 17C 40G variant of Aurora 2 over a range of 4-MUP concentrations. Spectra were measured in the presence of 500 μM DNA in a buffer containing 200 mM KCl, 1 mM $ZnCl_2$, 5% (v/v) DMSO, 50 mM HEPES, pH 7.4, and different concentrations of 4-MUP. (B) Graph showing the area under the curve of the first peak from the left in panel A. Data were fit using the Hill equation: Y=Bottom + (Top-Bottom)/(1+10^((LogEC50-X)*HillSlope)) using Prims10 software.

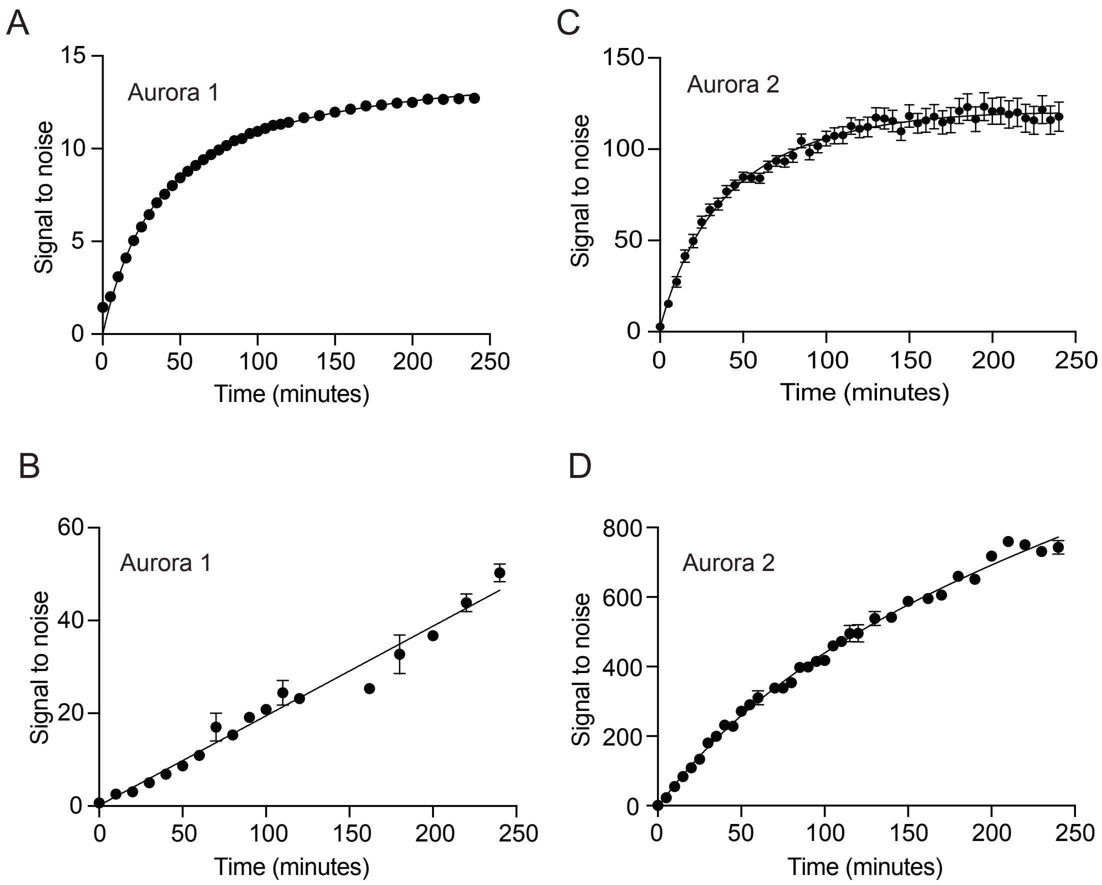

Supplementary Figure 8. Maximum signal to noise ratio of Aurora in continuous and discontinuous assays. (A) Assay in which Aurora 1 was mixed with 4-MUP and fluorescence was measured continuously using a plate reader. (B) Assay in which Aurora 1 was mixed with 4-MUP and time points were quenched with 20 μl of 1 M KOH before measuring fluorescence. (C) Same as panel A, but using Aurora 2. (D) Same as panel B, but using Aurora 2. Reactions were performed using 15 μM Aurora and 30 μM 4-MUP in a buffer containing 200 mM KCl, 1 mM $ZnCl_2$, 5% (v/v) DMSO, and 50 mM HEPES, pH 7.4, and fluorescence was measured using a TECAN Spark plate reader. Signal to noise ratio is defined as the rate of production of fluorescence in the presence of deoxyribozyme divided by the rate in the absence of deoxyribozyme. Points show the average values of three experiments, and error bars represent one standard deviation.

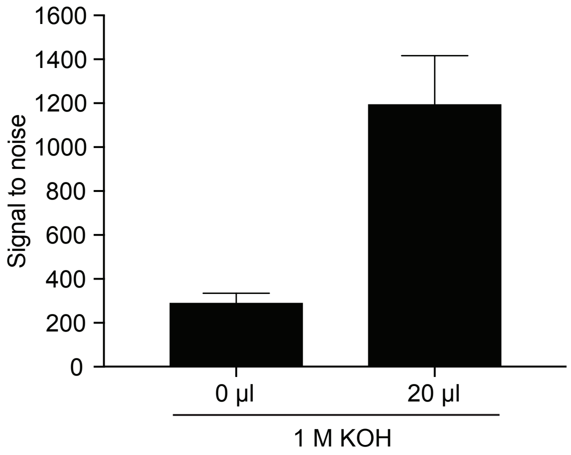

Supplementary Figure 9. Fluorescence of a synthetic standard. (A) Signal to noise ratio of fluorescence of synthetic 4-MU in the absence and presence of base. Samples contained 15 µM 4-MU or 15 µM 4-MUP and 200 mM KCl, 1 mM $ZnCl_2$, 5% (v/v) DMSO and 50 mM HEPES, pH 7.4. After incubating for 4 hours, either 0 µl or 20 µl of 1 M KOH was added and fluorescence was measured using a TECAN Spark plate reader. Signal to noise ratio is defined as the rate of fluorescence production in the presence of 4-MU divided by the rate of fluorescence production in the presence of 4-MUP. Columns show the average of at least three experiments, and error bars represent one standard deviation.

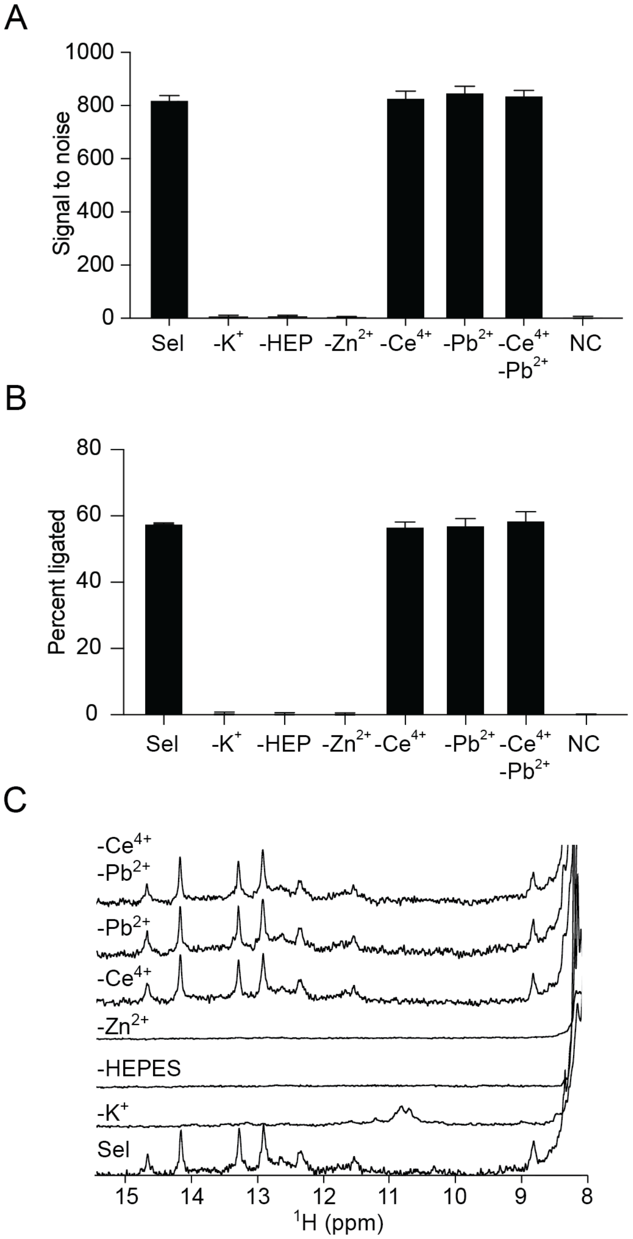

Supplementary Figure 10. Aurora requires potassium and zinc for activity and folding. (A) Signal to noise ratio of Aurora in a series of buffers in which different components were omitted. The original selection buffer (labeled “Sel”) contained 200 mM KCl, 1 μM CeO_2, 0.1 μM $PbCl_2$, 1 mM $ZnCl_2$, and 50 mM HEPES, pH 7.4. Reactions were performed using 15 μM Aurora and 30 μM 4-MUP. After incubating for 4 hours, 20 μl of 1 M KOH was added and fluorescence was measured using a TECAN Spark plate reader. (B) Same experiment as in panel A, but showing the percent of phosphorylated Aurora measured using the ligation assay. The percent ligated was determined after incubating 1 μM Aurora with 1 mM 4-MUP for 1 hour. (C) Proton NMR spectra of Aurora in the same buffers used in panel A. Spectra were measured using 300 μM Aurora and 450 μM 4-MUP. Signal to noise ratio is defined as the rate of fluorescence production in the presence of deoxyribozyme divided by the rate of fluorescence production in the absence of deoxyribozyme. Columns show the values from at least three experiments, and error bars represent one standard deviation. Experiments were performed using Aurora 2.

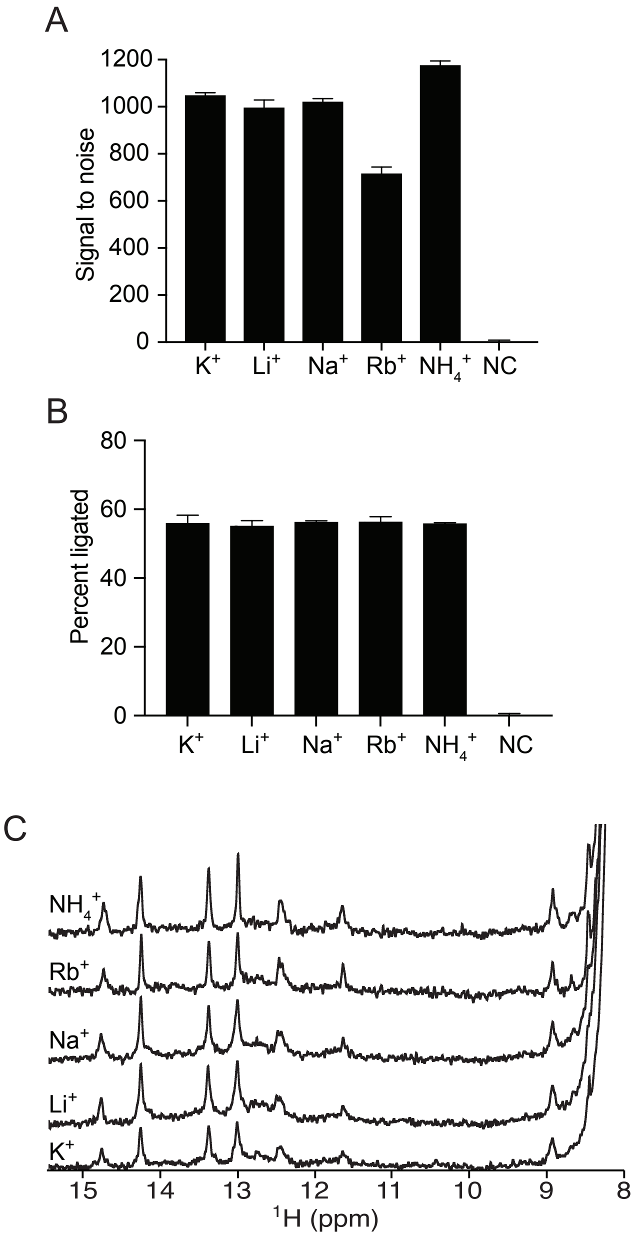

Supplementary Figure 11. Aurora is active in a wide range of monovalent metal ions. (A) Replacement of potassium with other monovalent metal ions. Buffers contained the indicated monovalent cation at a concentration of 200 mM, 1 mM $ZnCl_2$, 5% (v/v) DMSO, and 50 mM HEPES, pH 7.4. Reaction were performed using 15 μM Aurora and 30 μM 4-MUP. After incubating for 4 hours, 20 μl of 1 M KOH was added and fluorescence was measured using a TECAN Spark plate reader. (B) Same experiment as in panel A, but showing the percent of phosphorylated Aurora measured using the ligation assay. The percent ligated was determined after incubating 1 μM Aurora with 1 mM 4-MUP for 1 hour. (C) Proton NMR spectra of Aurora in the same buffers used in panel A. Spectra were measured using 300 μM Aurora and 450 μM 4-MUP. Signal to noise ratio is defined as the rate of fluorescence production in the presence of deoxyribozyme divided by the rate of fluorescence production in the absence of deoxyribozyme. Columns show the values from at least three experiments, and error bars represent one standard deviation. Experiments were performed using Aurora 2.

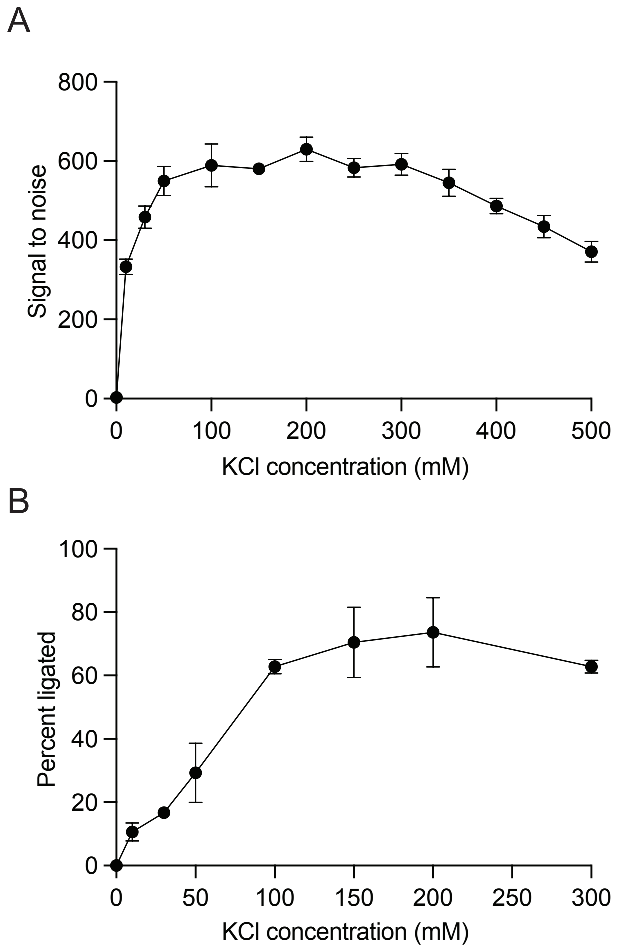

Supplementary Figure 12. Dependence of Aurora activity on KCl concentration. (A) Signal to noise ratio of Aurora over a range of potassium concentrations. Buffers contained the indicated concentration of KCl, 1 mM $ZnCl_2$, 5% (v/v) DMSO, and 50 mM HEPES, pH 7.4. Reactions were performed using 15 µM Aurora and 30 µM 4-MUP. After incubating for 4 hours, 20 µl of 1 M KOH was added and fluorescence was measured using a TECAN Spark plate reader. (B) Same experiment as in panel A, but showing the percent of phosphorylated Aurora measured by the ligation assay. The percent ligated was determined after incubating 1 µM Aurora with 1 mM 4-MUP for 1 hour. Signal to noise ratio is defined as the rate of fluorescence production in the presence of deoxyribozyme divided by the rate of fluorescence production in the absence of deoxyribozyme. Points show the average of three experiments, and error bars represent one standard deviation. Experiments were performed using Aurora 2.

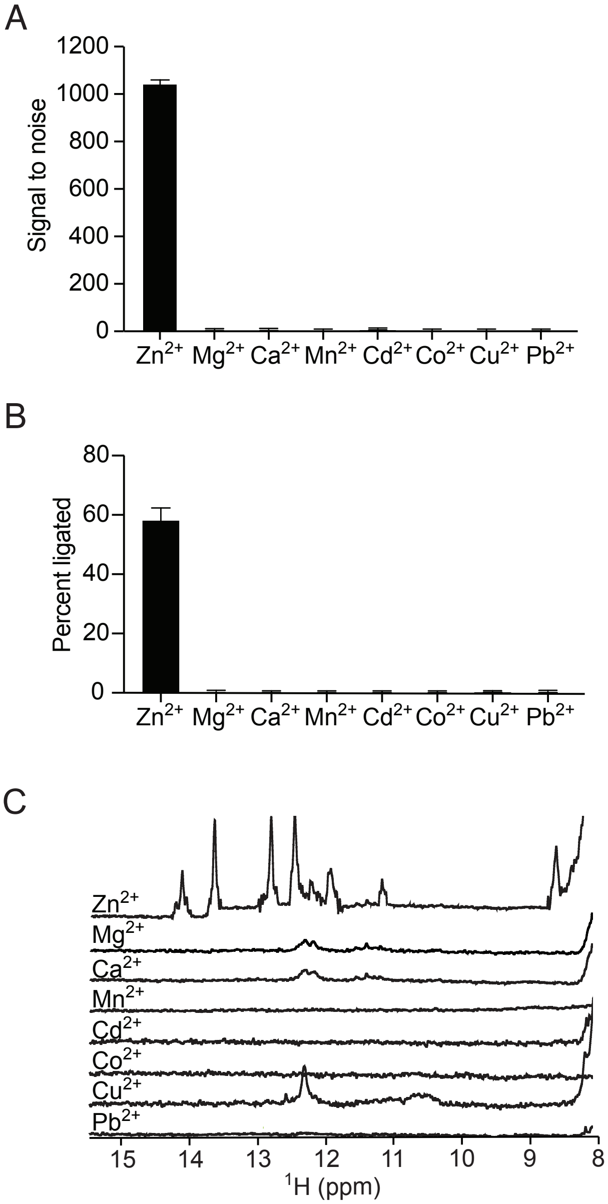

Supplementary Figure 13. Aurora is active in the presence of zinc, but not in the presence of other divalent metal ions. (A) Replacement of zinc with other divalent metal ions. Buffers contained 1 mM of the indicated divalent cation, 200 mM KCl, 5% (v/v) DMSO, and 50 mM HEPES, pH 7.4. Reaction were performed using 15 μM Aurora and 30 μM 4-MUP. After incubating for 4 hours, 20 μl of 1 M KOH was added and fluorescence was measured using a TECAN Spark plate reader. (B) Same experiment as in panel A, but showing the percent of phosphorylated Aurora measured using the ligation assay. The percent ligated was determined after incubating 1 μM Aurora with 1 mM 4-MUP for 1 hour. (C) Proton NMR spectra of Aurora in the same buffers used in panel A. Spectra were measured using 300 μM Aurora and 450 μM 4-MUP. Signal to noise ratio is defined as the rate of fluorescence production in the presence of deoxyribozyme divided by the rate of fluorescence production in the absence of deoxyribozyme. Columns show the values from at least three experiments, and error bars represent one standard deviation. Experiments were performed using Aurora 2.

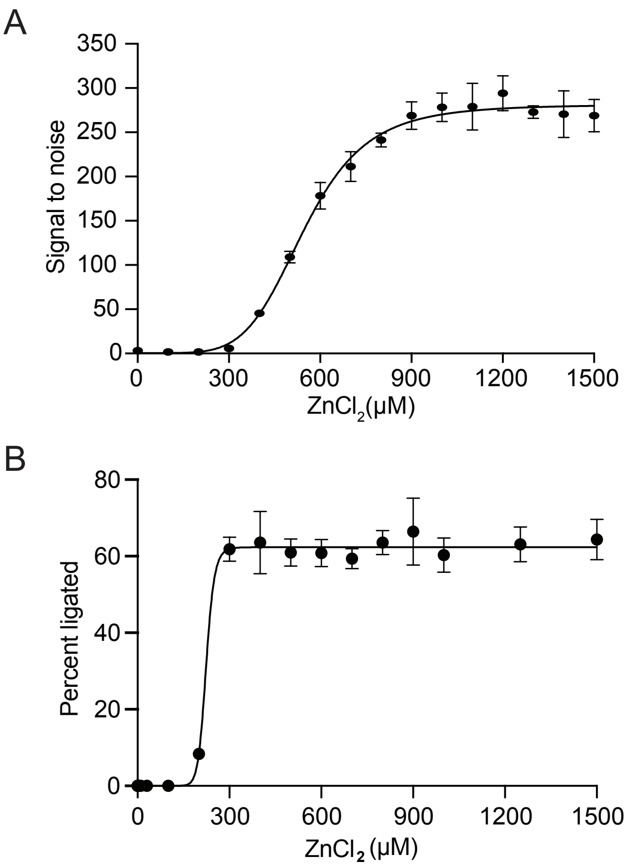

Supplementary Figure 14. Cooperative effect of zinc on Aurora catalytic activity. (A) Signal to noise ratio of Aurora as a function of zinc concentration. Buffers contained the indicated concentration of $ZnCl_2$, 200 mM KCl, 5% (v/v) DMSO, and 50 mM HEPES, pH 7.4. Reactions were performed using 15 μM Aurora and 30 μM 4-MUP. After incubating for 4 hours, 20 μl of 1 M KOH was added and fluorescence was measured using a TECAN Spark plate reader. (B) Same experiment as in panel A, but showing the percent of phosphorylated Aurora measured using the ligation assay. The percent ligated was determined after incubating 1 μM Aurora with 1 mM 4-MUP for 1 hour. Signal to noise ratio is defined as the rate of fluorescence production in the presence of deoxyribozyme divided by the rate of fluorescence production in the absence of deoxyribozyme. Points show the average of at least three experiments, and error bars represent one standard deviation. Experiments were performed using Aurora 2.

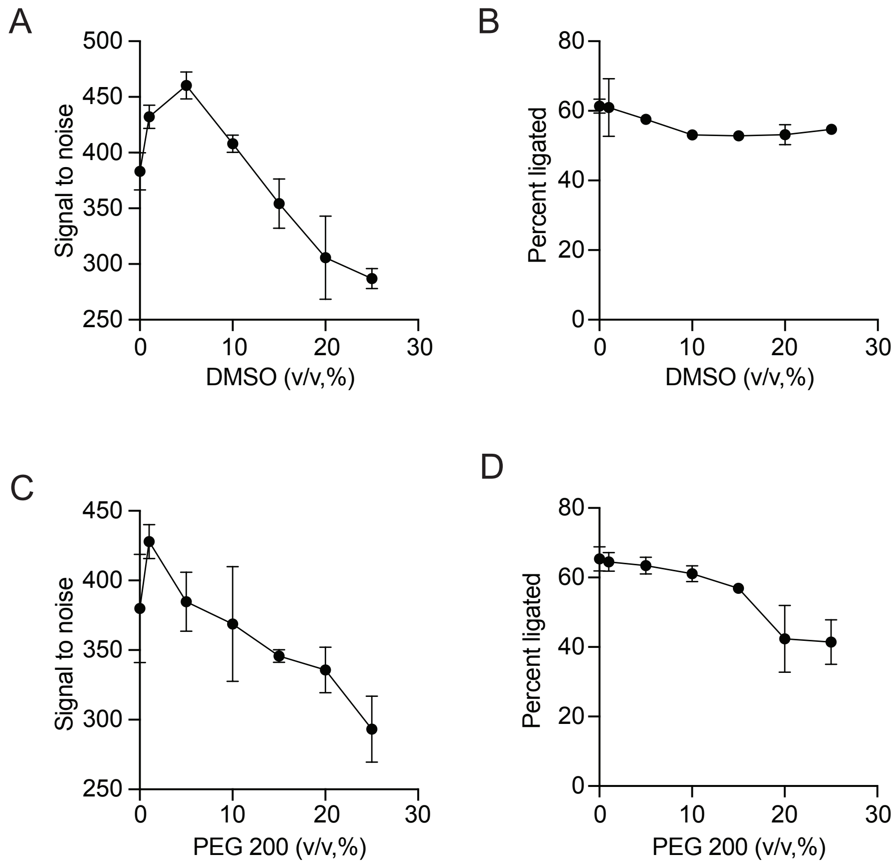

Supplementary Figure 15. Effect of molecular crowding agents and organic solvents on the catalytic activity of Aurora. A) Signal to noise ratio of Aurora over a range of DMSO concentrations. Buffers contained the indicated concentration of DMSO, 200 mM KCl, 1 µM CeO_2, 0.1 µM $PbCl_2$, 1 mM $ZnCl_2$, and 50 mM HEPES, pH 7.4. Reactions were performed using 15 µM Aurora and 30 µM 4-MUP. After incubating for 4 hours, 20 µl of 1 M KOH was added and fluorescence was measured using a TECAN Spark plate reader. (B) Same experiment as in panel A, but showing the percent of phosphorylated Aurora measured using the ligation assay. The percent ligated was determined after incubating 1 µM Aurora with 1 mM 4-MUP for 1 hour. (C) Signal to noise ratio of Aurora over a range of PEG 200 concentrations. Buffers contained the indicated concentration of PEG 200, 200 mM KCl,1 µM CeO_2, 0.1 µM $PbCl_2$, 1 mM $ZnCl_2$, and 50 mM HEPES, pH 7.4. Reactions were performed using 15 µM Aurora and 30 µM 4-MUP. After incubating for 4 hours, 20 µl of 1 M KOH was added and fluorescence was measured using a TECAN Spark plate reader. (D) Same experiment as in panel C, but showing the percent of phosphorylated Aurora measured using the ligation assay. The percent ligated was determined after incubating 1 µM Aurora with 1 mM 4-MUP for 1 hour. Signal to noise ratio is defined as the rate of fluorescence production in the presence of deoxyribozyme divided by the rate of fluorescence production in the absence of deoxyribozyme. Points show the values from three experiments, and error bars represent one standard deviation. Experiments were performed using Aurora 2.

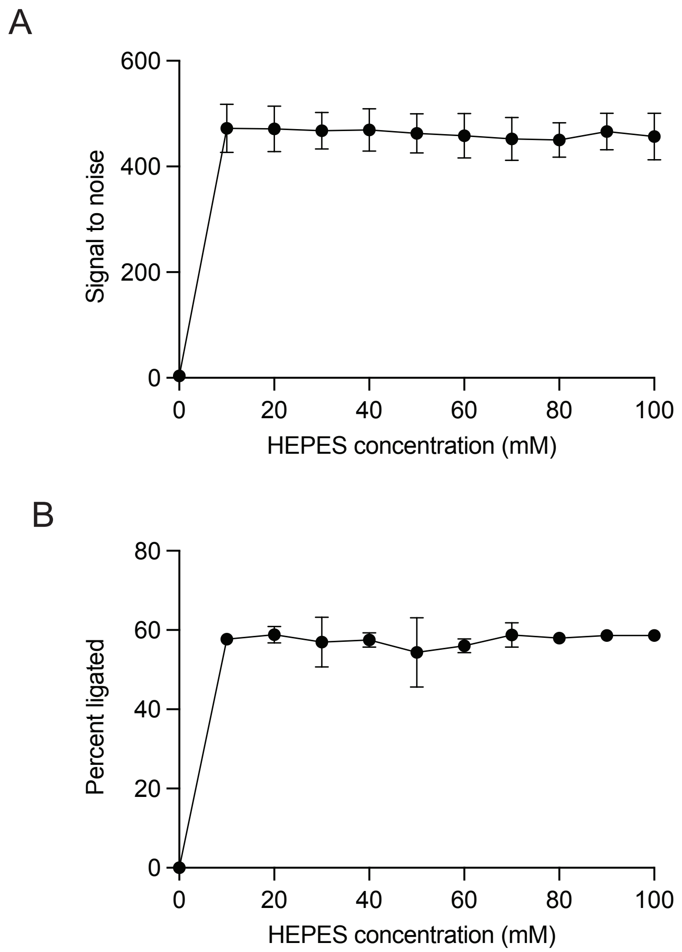

Supplementary Figure 16. Dependence of Aurora activity on HEPES concentration. (A) Signal to noise ratio of Aurora as a function of HEPES concentration. Buffers contained the indicated concentration of HEPES, pH 7.4, 200 mM KCl, 1 mM $ZnCl_2$, and 5% (v/v) DMSO. Reactions were performed using 15 µM Aurora and 30 µM 4-MUP. After incubating for 4 hours, 20 µl of 1 M KOH was added and fluorescence was measured using a TECAN Spark plate reader. (B) Same experiment as in panel A, but showing the percent of phosphorylated Aurora measured using the ligation assay. The percent ligated was determined after incubating 1 µM Aurora with 1 mM 4-MUP for 1 hour. Signal to noise ratio is defined as the rate of fluorescence production in the presence of deoxyribozyme divided by the rate of fluorescence production in the absence of deoxyribozyme. Points show the average of three experiments, and error bars represent one standard deviation. Experiments were performed using Aurora 2.

A

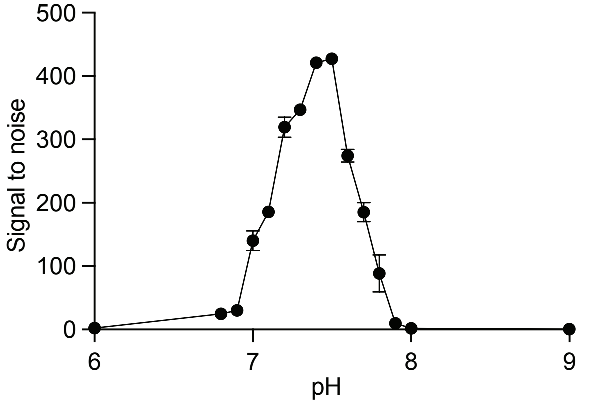

B

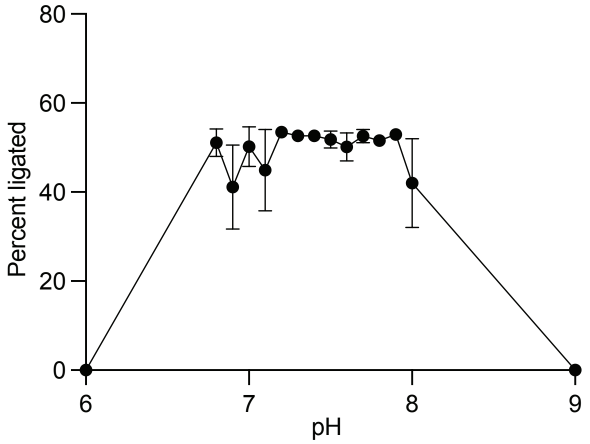

Supplementary Figure 17. Effect of pH on Aurora catalytic activity. (A) Signal to noise ratio of Aurora as a function of pH. Buffers contained 50 mM HEPES at the indicated pH, 200 mM KCl, 1 mM $ZnCl_2$, and 5% (v/v) DMSO. Reactions were performed using 15 μM Aurora and 30 μM 4-MUP. After incubating for 4 hours, 20 μl of 1 M KOH was added and fluorescence was measured using a TECAN Spark plate reader. (B) Same experiment as in panel A, but showing the percent of phosphorylated Aurora measured using the ligation assay. The percent ligated was determined after incubating 1 μM Aurora with 1 mM 4-MUP for 1 hour. Signal to noise ratio is defined as the rate of fluorescence production in the presence of deoxyribozyme divided by the rate of fluorescence production in the absence of deoxyribozyme. Points show the average of at least three experiments, and error bars represent one standard deviation. Experiments were performed using Aurora 2.

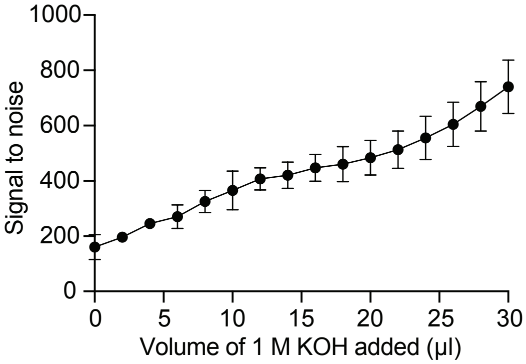

Supplementary Figure 18. The signal generated by Aurora can be enhanced by adding base before measuring fluorescence. Effect of pH adjustment on the signal to noise ratio of Aurora. Buffer contained 200 mM KCl, 1 µM $Ce(NO_3)_4$, 0.1 µM $PbCl_2$, 1 mM $ZnCl_2$, and 50 mM HEPES, pH 7.4. Reactions were performed using 15 µM Aurora and 30 µM 4-MUP. After incubating for 4 hours, the indicated amount of M KOH was added and fluorescence was measured using a TECAN Spark plate reader. Signal to noise ratio is defined as the rate of fluorescence production in the presence of deoxyribozyme divided by the rate of fluorescence production in the absence of deoxyribozyme. Points show the values from at least three experiments, and error bars represent one standard deviation. Experiments were performed using Aurora 2.

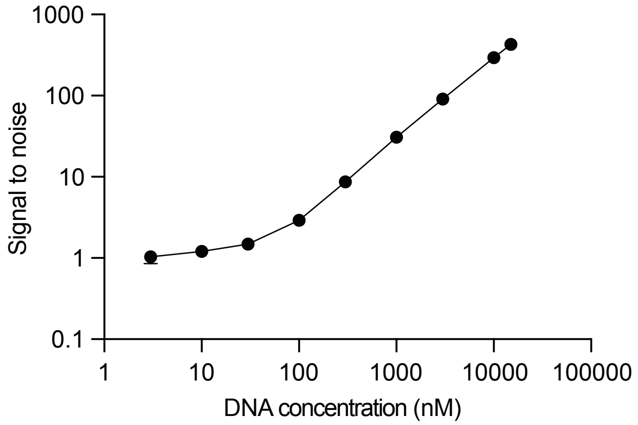

Supplementary Figure 19. Detection limit of Aurora. Signal to noise ratio of fluorescence production over a range of concentrations of Aurora. Reactions were performed using the indicated concentration of Aurora and 30 μM 4-MUP in a buffer containing 200 mM KCl, 1 mM $ZnCl_2$, 5% (v/v) DMSO, and 50 mM HEPES, pH 7.4. After incubating for 4 hours, 20 μl of 1 M KOH was added and fluorescence was measured using a TECAN Spark plate reader. Signal to noise ratio is defined as the rate of fluorescence production in the presence of deoxyribozyme divided by the rate of fluorescence production in the absence of deoxyribozyme. Points show the average of three experiments, and error bars represent one standard deviation. Experiments were performed using Aurora 2.

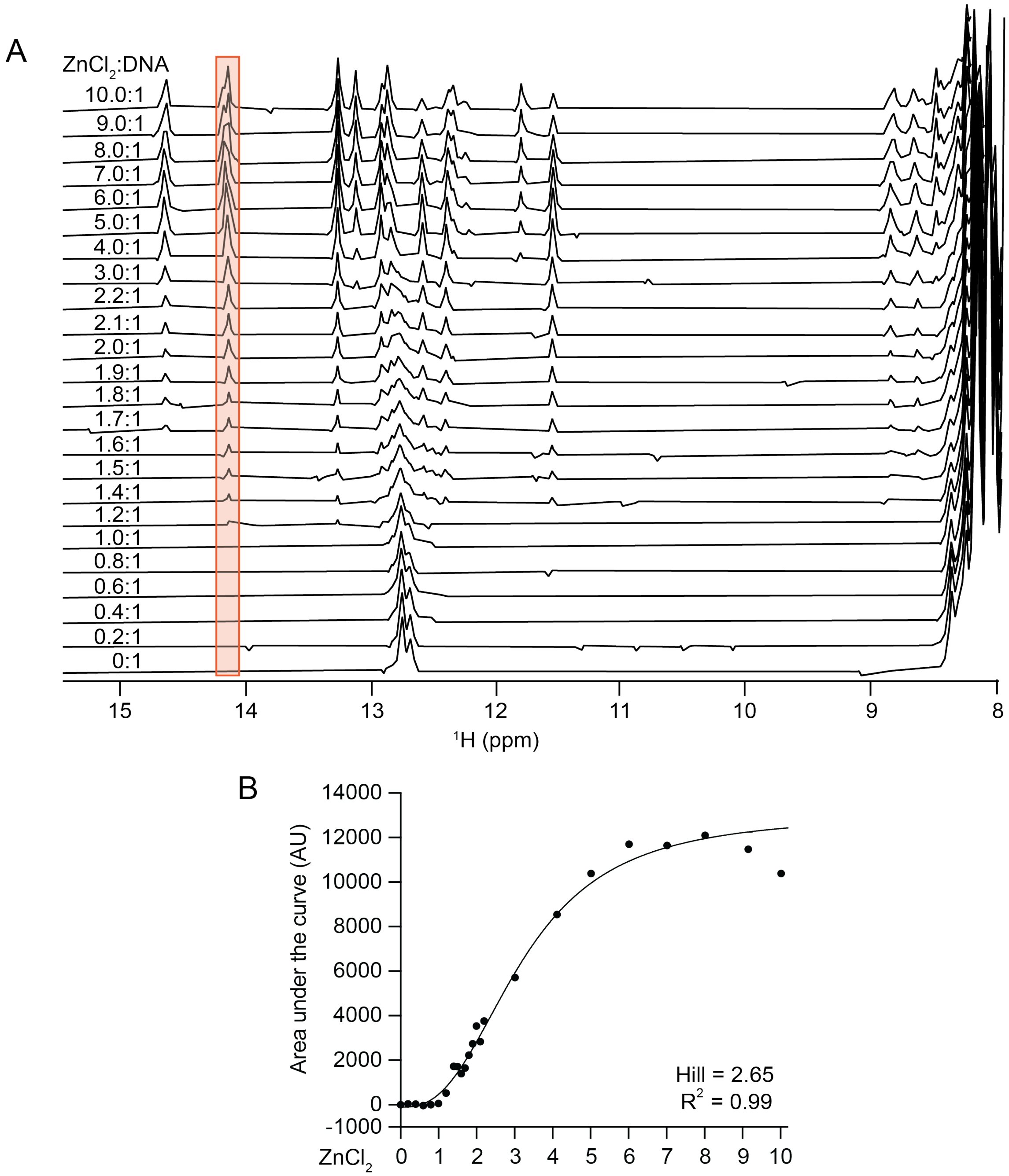

Supplementary Figure 20. Cooperative effect of zinc on Aurora folding. (A) Proton NMR spectra of the 17C 40G variant of Aurora over a series of concentrations of $ZnCl_2$. Spectra were measured in the presence of 500 μM DNA, 750 μM 4-MUP, 200 mM KCl, 5% (v/v) DMSO, and 50 mM HEPES, pH 7.4, and different concentrations of $ZnCl_2$. (B) Graph showing the area under the curve of the second peak from the left (indicated with an orange box) in panel A. Data were fit with the Hill equation: Y=Bottom + (Top-Bottom)/(1+10^((LogEC50-X)*HillSlope)) using Prims10 software.

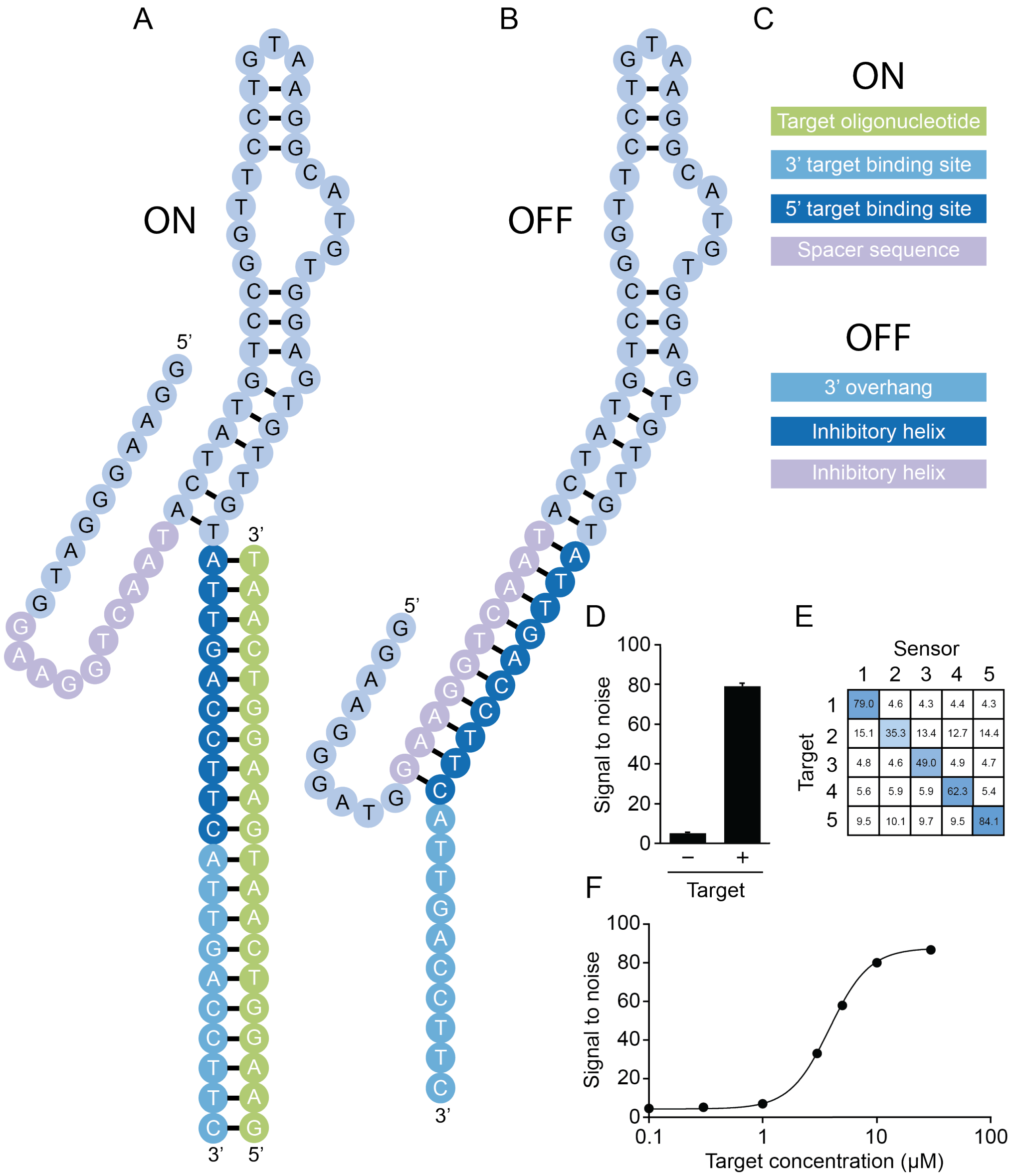

Supplementary Figure 21. Programmable oligonucleotide sensor based on Aurora. To make such a sensor, part of the target sequence (shown in purple) is inserted between nucleotides 10 and 11 of Aurora, and the reverse complement of the full target sequence (shown in dark blue and medium blue) is added to the 3' end of Aurora. A) In the ON version of the sensor, the target oligonucleotide (shown in green) base pairs to the 3' end of Aurora (shown in dark blue and medium blue). This prevents formation of an inhibitory helix and enables catalytic activity. B) In the OFF version of the sensor, the sequences shown in purple and dark blue form an inhibitory helix that inactivates Aurora, presumably by increasing the distance between the reaction site (the 5' hydroxyl group) and the catalytic core. Note that this interaction cannot occur in the presence of the target oligonucleotide C) Roles played by different parts of the sensor in the presence (above, ON), and absence (below, OFF) of the target oligonucleotide. D) Production of fluorescence in the absence (left) and presence (right) of the target oligonucleotide. E) Specificity of the sensor. Five different sensors were generated, each of which was designed to detect a different target oligonucleotide. Each square represents a different sensor/target combination, and signal to noise ratios are indicated with both numbers and colors. F) Detection limit of the sensor. Reactions were incubated for four hours in the presence of the indicated

concentration of the target oligonucleotide, and after quenching with base, fluorescence was measured using a plate reader. See Supplementary Table 1 for the sequences of deoxyribozymes used in the experiments described in this figure.

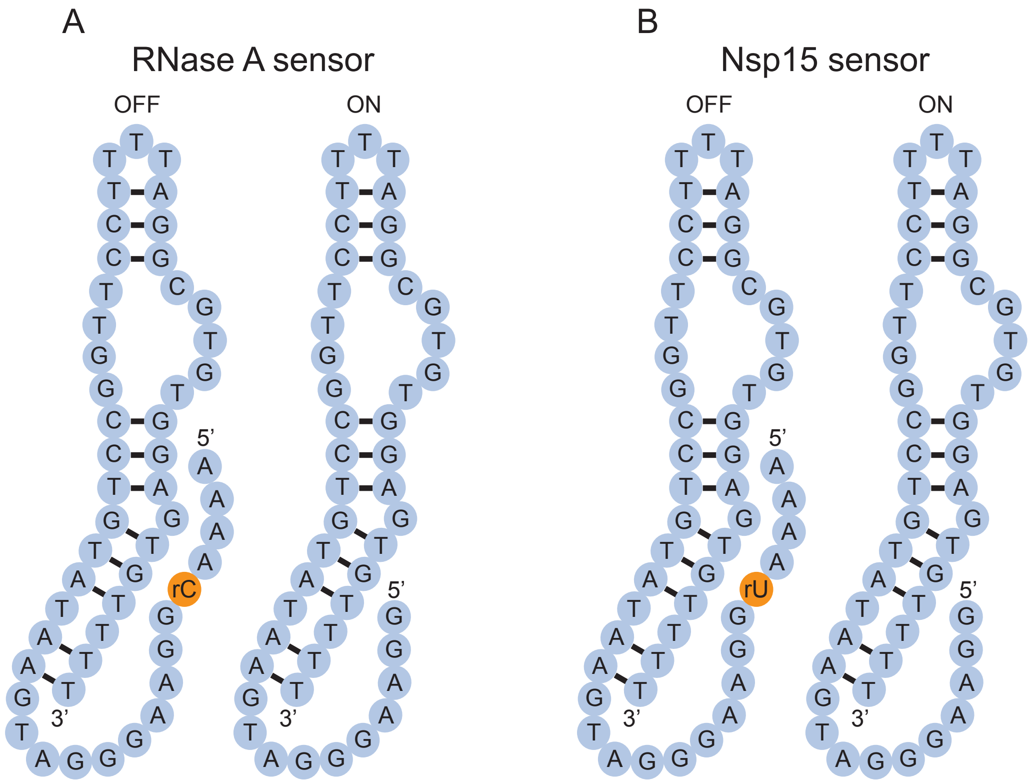

Supplementary Figure 22. Design of a ribonuclease sensor based on Aurora. Left: sensor activated by RNase A. Right: sensor activated by Nsp15. The RNA base in each sensor is shown in orange.

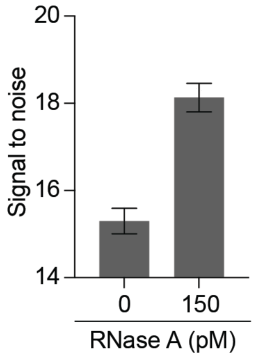

Supplementary Figure 23. Detection limit of the Aurora RNase sensor for RNase A. Signal to noise ratio is defined as the rate of fluorescence production in the presence of sensor divided by the rate of fluorescence production in the absence of sensor. Columns show the values from at least three experiments, and error bars represent one standard deviation. See Supplementary Figure 22 and Supplementary Table 1 for the sequence of the Aurora sensor used in this experiment and the methods section of the manuscript for more details about the protocol.

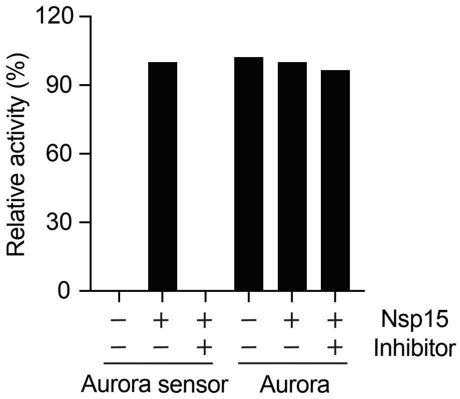

Supplementary Figure 24. Activation of an Aurora sensor by Nsp15. Left: an inactive form of Aurora is activated by Nsp15, but not when an RNase inhibitor is present. Right: neither Nsp15 nor inhibitor affects the catalytic activity of unmodified Aurora. Nsp15 cleavage reactions were performed using 25 μM of either Aurora sensor or Aurora 2, 400 nM Nsp15, and either 0 μM or 200 μM inhibitor (See Figure 4 of the manuscript for the chemical structure of this inhibitor) in a buffer containing 50 mM KCl, 20 mM HEPES pH 7.4, 5 mM $MnCl_2$, and 0.003% (v/v) Tween20 in a volume of 20 μl. After incubating at room temperature for 1 hour, 80 μl of a new buffer was added to initiate Aurora catalysis. Final conditions were 5 μM of either Aurora sensor or Aurora 2, 80 nM Nsp15, and either 0 μM or 40 μM inhibitor in a buffer containing 200 mM KCl, 1 mM $ZnCl_2$, 5% (v/v) DMSO, and 50 mM HEPES, pH 7.4. After incubating for 4 hours, fluorescence was measured using a TECAN Spark plate reader. See Supplementary Figure 22 and Supplementary Table 1 for the sequence of the Aurora sensor used in this experiment and the methods section of the manuscript for more details about the protocol.

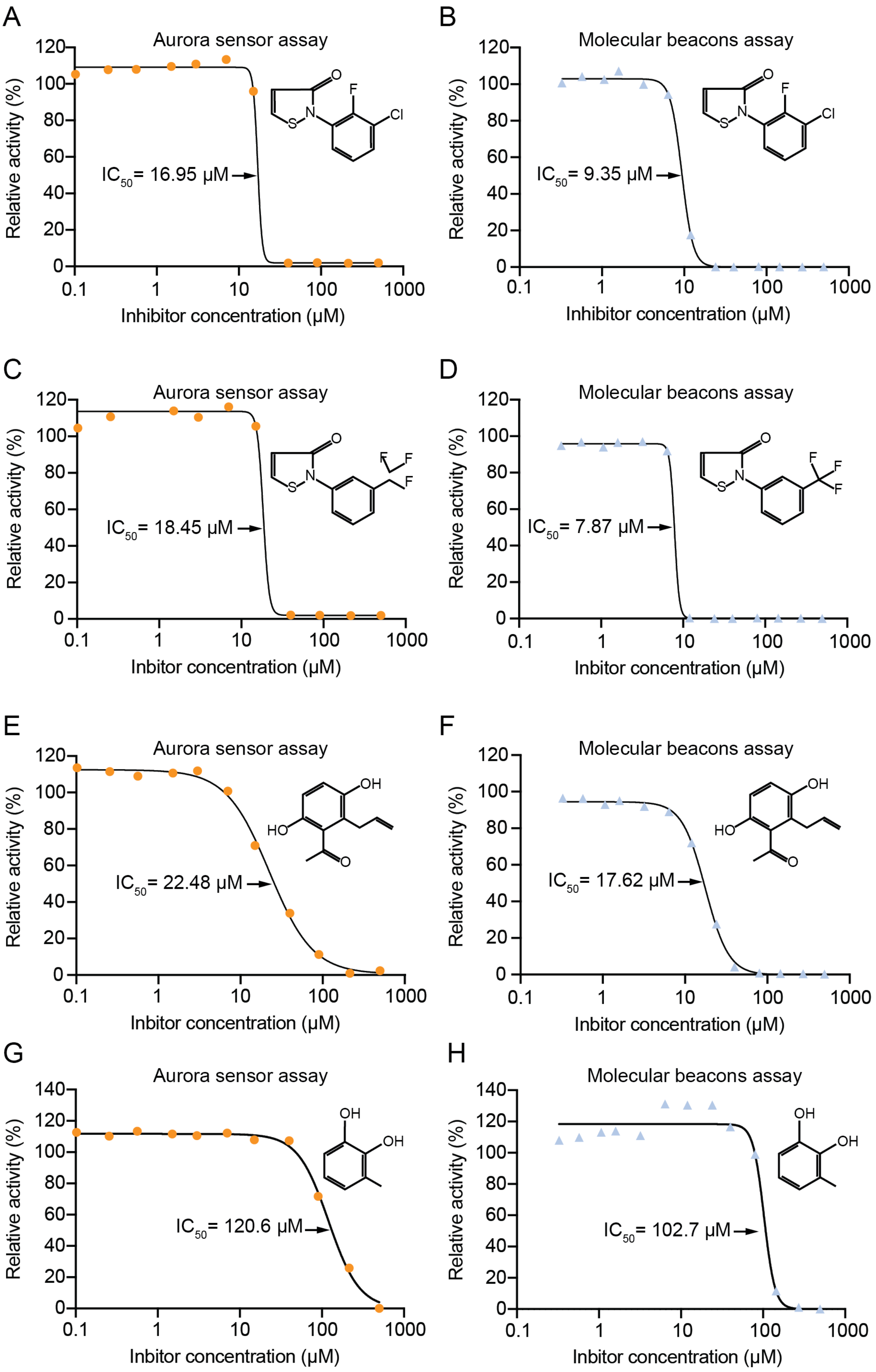

A
Aurora sensor assay
Relative activity (%)
IC50= 16.95 μM
Inhibitor concentration (μM)
B
Molecular beacons assay
Relative activity (%)
IC50= 9.35 μM
Inhibitor concentration (μM)
C
Aurora sensor assay
Relative activity (%)
IC50= 18.45 μM
Inbitor concentration (μM)
D
Molecular beacons assay
Relative activity (%)
IC50= 7.87 μM
Inbitor concentration (μM)
E
Aurora sensor assay
Relative activity (%)
IC50= 22.48 μM
Inbitor concentration (μM)
F
Molecular beacons assay
Relative activity (%)
IC50= 17.62 μM
Inbitor concentration (μM)
G
Aurora sensor assay
Relative activity (%)
IC50= 120.6 μM
Inbitor concentration (μM)
H
Molecular beacons assay
Relative activity (%)
IC50= 102.7 μM
Inbitor concentration (μM)

Supplementary Figure 25. IC50 values for Nsp15 inhibitors isolated in the high-throughput screen. Experiments were performed using an Aurora sensor (left column) and a FRET assay (right column).
